# Revision of the Neotropical caddisfly genus *Itauara* Müller, 1888 (Trichoptera, Glossosomatidae)

**DOI:** 10.3897/zookeys.114.1405

**Published:** 2011-06-30

**Authors:** Desiree R. Robertson, Ralph W. Holzenthal

**Affiliations:** 1 University of Minnesota, Department of Entomology, 1980 Folwell Ave., Room 219, St. Paul, Minnesota 55108, U.S.A.; 2 Frostburg State University, Biology Department, 101 Braddock Rd., Frostburg, Maryland 21532, U.S.A.

**Keywords:** Trichoptera, Glossosomatidae, Protoptilinae, systematics, taxonomy, *Itauara*, new species, Neotropical, male genitalia

## Abstract

Systematics of the genus *Itauara* Müller, 1888 are reviewed. A generic diagnosis, illustrations, and descriptions are provided for males. The genus can be identified by several features of the male genitalia including an extremely reduced phallobase and a phallic apparatus that consists of a sclerotized dorsal sheath covering a very membranous ventral portion. A total 18 species are described as new: *Itauara alexanderi* **sp. n.**(Brazil), *Itaura bidentata* **sp. n.** (Guyana), *Itaura blahniki* **sp. n.** (Brazil) *Itaura charlotta* **sp. n.** (Brazil), *Itaura emilia* **sp. n.** (Brazil), *Itaura flinti* **sp. n.** (Brazil), *Itaura guyanensis* **sp. n.** (Guyana), *Itaura jamesii* **sp. n.** (Brazil), *Itaura julia* **sp. n.** (Brazil), *Itaura lucinda* **sp. n.** (Brazil), *Itaura ovis* **sp. n.** (Guyana, Venezuela), *Itaura peruensis* **sp. n.** (Peru), *Itaura rodmani* **sp. n.** (Brazil), *Itaura simplex* **sp. n.** (Brazil), *Itaura spiralis* **sp. n.** (Guyana), *Itaura stella* **sp. n.** (Brazil), *Itaura tusci* **sp. n.** (Brazil), and *Itaura unidentata* **sp. n.** (Guyana). These additions bring the total fauna of *Itauara* to 22 species.

## Introduction

The genus *Itauara* Müller, 1888, belongs to the saddle-, or tortoise-case making caddisfly family Glossosomatidae. The name *Itauara* comes from the Tupi-Guarani language and roughly translates to “born from rock,” likely referring to glossosomatid larval cases, often found conspicuously on the surface of submerged rocks. *Itauara* larvae construct rather loose, easily deformable cases of large and small grains of sand that are vaulted dorsally, and almost flat ventrally (Angrisano 1993). In southeastern Brazil and surrounding regions in Argentina and Uruguay, larvae are known to occur in sandy bottom streams with scarce vegetation where they attach their cases to Characeae algae (Angrisano 1993).

**Figure 1.**
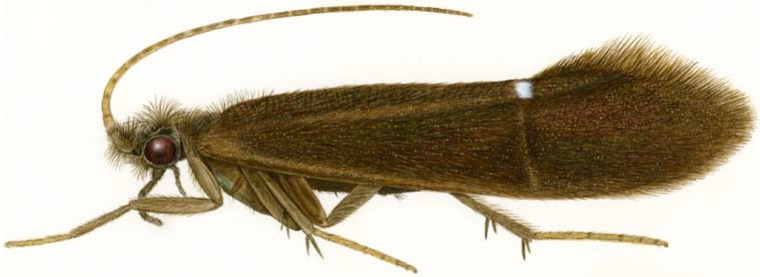
Adult, lateral view, *Itauara julia* sp. n.

Like other members of Protoptilinae, *Itauara* adults are minute, usually less than 3 mm in size. Their wings are brownish and may have a conspicuous white spot at the arculus or transverse line along the anastomosis (Fig. 1). *Itauara* are rather rare in occurrence; indeed, several of the new species described here are known from only a single specimen. The 4 known species of *Itauara* occur only in South America, with 3 of these [*Itaura brasiliana* (Mosely, 1939), *Itaura guarani* (Angrisano, 1993), and *Itaura plaumanni* (Flint, 1974)] endemic to southeastern Brazil and surrounding regions of Argentina and Uruguay. A fourth species, *Itaura amazonica* (Flint, 1971), is known from Amazonas state, Brazil.

A recent survey of the Trichoptera of southeastern Brazil by researchers at the University of Minnesota yielded numerous new species of *Itauara* and impetus for this study. Dr. Oliver S. Flint, National Museum of Natural History, Smithsonian Institution, generously provided several additional undescribed species from various locales in South America for inclusion in this study. In this paper, we determine the homologies and establish standardized terminology of the male genitalic structures among species. Additionally, we re-describe and illustrate the 4 known *Itauara* species and describe 18 new species from southeastern Brazil, Peru, Guyana, and Venezuela, bringing the total number of species to 22. These species are here assigned to 2 species groups and listed in Table 1. Finally, we provide a key to the males of *Itauara*.

**Table 1.** *Itauara* species groups (Trichoptera: Glossosomatidae: Protoptilinae).

*Itauara amazonica* species group
*Itauara alexanderi* sp. n.
*Itauara amazonica* (Flint, 1971)
*Itauara bidentata* sp. n.
*Itauara emilia* sp. n.
*Itauara guyanensis* sp. n.
*Itauara jamesii* sp. n.
*Itauara lucinda* sp. n.
*Itauara ovis* sp. n.
*Itauara peruensis* sp. n.
*Itauara spiralis* sp. n.
*Itauara stella* sp. n.
*Itauara unidentata* sp. n.
*Itauara brasiliana* species group
*Itauara blahniki* sp. n.
*Itauara brasiliana* (Mosely, 1954)
*Itauara charlotta* sp. n.
*Itauara flinti* sp. n.
*Itauara guarani* (Angrisano, 1993)
*Itauara julia* sp. n.
*Itauara plaumanni* (Flint, 1974)
*Itauara rodmani* sp. n.
*Itauara simplex* sp. n.
*Itauara tusci* sp. n.

## Material and methods

### Specimen preparation and observation

To observe certain structural features of the male genitalia, soft tissues were cleared using a lactic acid method outlined in detail by Holzenthal and Anderson (2004) and Blahnik et al. (2007). For some specimens, the entire individual was cleared (after removing the wings) to more easily observe external structures obscured by setae, such as thoracic warts. Specimens that were over-cleared or lightly sclerotized were stained. Such specimens were immersed in a small watch-glass containing Chlorazole Black E (Sigma Chemical Co.) dissolved in glycerin for 15 minutes to several hours, depending on the size and condition of the specimen. Stained specimens were then rinsed in distilled water to remove any excess stain. Specimens were examined in a small watch-glass containing glycerin using an Olympus SZX12 dissecting microscope or Olympus BX41 compound microscope. To observe wing venation, wing mounts of each species were prepared following the protocols of Blahnik and Holzenthal (2004).

### Illustrations, descriptions, and identification key

Pencil sketches of the male genitalia were completed using either an optical grid on a dissecting microscope, or *camera lucida* (drawing tube) mounted on a compound microscope. Pencil sketches were scanned digitally, and then placed as a template layer in Adobe Illustrator® for final rendering. Wing preparations were digitally photographed using a Leica EC3 digital camera mounted on an Olympus SZX12 dissecting microscope. Digital images were then placed as a template layer in Adobe Illustrator® for final rendering. Descriptions of species and generation of the identification key were facilitated by using the software package DELTA (DEscriptive Language for Taxonomy) (Dallwitz 1980; Dallwitz et al. 1993 onwards; 1999 onwards).

Females, with similar size and coloration as males that were collected at the same time and locality, are listed as paratypes under the material examined for some species. Previous experience has shown that having presumptively associated female specimens may be useful for future associative studies. However, since there is some uncertainty of association, we have deferred descriptions of females.

### Morphological terminology

Morphological terminology for male genitalia was adapted from Blahnik and Holzenthal (2006; 2008), Holzenthal (2004), Holzenthal and Blahnik (2006), and Morse (1988). Terminology for specific structures of male genitalia, as homologized in this study, is indicated in Figures 4–25. Wing venation terminology follows the Comstock-Needham system as interpreted by Ross (1956) and Schmid (1998).

### Depositories

Types and additional material examined for this study are deposited at the British Museum of Natural History, London, UK (BMNH); the National Museum of Natural History, Washington, DC, USA (NMNH), the Museo Argentino de Ciencias Naturales Bernardino Rivadavia, Buenas Aires, Argentina (MACN), the Museu de Zoologia, Universidade de São Paulo, São Paulo, Brazil (MZUSP), and the University of Minnesota Insect Collection, Saint Paul, USA (UMSP). All specimens or lot of alcohol specimens examined in this study were affixed with a barcode label with a unique 9 digit alphanumeric code starting with the prefix UMSP. This prefix indicates that the specimen has been databased at UMSP, but it is not meant to imply possession by UMSP. Specimen-level taxonomic, locality, and other information, are stored in the University of Minnesota Insect Collection Biota Trichoptera Database using the software program Biota (Colwell 2003), and can be accessed at http://www.entomology.umn.edu/museum/databases/BIOTAdatabase.html.

## Systematics

Until recently, the generic status of *Itauara* was uncertain. Müller (1888) first used the name *Itauara* in a discussion of larval morphology, but he did not include any species or illustrations. In a later, posthumous work (Müller 1921), he provided sketches of the female forewing venation and some larval structures. Ulmer (1957) thought that Müller’s illustrations resembled those of other genera in Protoptilinae of South American origin, and suggested that the larvae be split into different species and perhaps even different genera. He also noted that Müller’s forewing illustration completely matched that of *Antoptila brasiliana* Mosely, 1939 (Ulmer 1957). In his studies of Trichoptera collected from the Amazon, Marlíer (1964) later described some Protoptilinae larvae and female pupae and attributed them to *Itauara*. However, since the pupae were all females and the wings were not in a condition to adequately observe wing venation, Marlíer (1964) declined to provide a species name. Later, Flint (1971, 1974) described 2 new *Antoptila* species from the Amazon and southeastern Brazil. Angrisano (1993) described the female, larvae and pupae of *Antoptila brasiliana* Mosely, 1939 and males and females of *Antoptila plaumanni* Flint, 1974 and another new species *Antoptila guarani* Angrisano, 1993. Subsequently, based on similarities in wing venation and of cases and larval morphology, Flint et al. (1999) synonymized *Itauara* with *Antoptila* Mosely 1939, designated *Antoptila brasiliana* as the type species, and transferred the 3 other known species of *Antoptila* to *Itauara*.

In a recent phylogenetic analysis of the entire protoptiline subfamily, *Itauara* was recovered as a monophyletic group with strong support (Robertson 2010). The presence of a dorsal sheath-like phallicata was identified as a unique synapomorphy of *Itauara* (Robertson 2010). Although members of this genus have superficially similar male genitalia, certain structures are not homologous. For example, the type species *Itaura brasiliana* (Mosely, 1939) has 2 pairs of curious elongate, seta-like processes on sternum IX. The 3 additional species placed in the genus have similarly looking elongate ventral processes, yet they are not the same as in the type species; these processes are parameres, and arise directly from the phallobase or endotheca, rather than sternum IX.

### Genus Itauara

Müller,  1888

http://species-id.net/wiki/Itauara

Itauara  Müller, 1888: 275 [Type species: *Antoptila brasiliana* Mosely, 1939, subsequent selection by Flint, Holzenthal, and Harris 1999].
Antoptila  Mosely, 1939: 219 [Type species: *Antoptila brasiliana* Mosely, 1939, original designation] Flint, Holzenthal, and Harris 1999, to synonymy.

#### Notes

The genus *Itauara* can be identified by features of the male genitalia. The phallic apparatus consists of a sclerotized dorsal sheath covering a very membranous ventral portion, an apparent posterior extension of the phallobase or phallicata. Rarely, the phallicata is tubular or separated from the phallobase by a membranous portion. In some species, this sclerotized dorsal sheath seems to detach from the ventral membrane apically to reveal a single dorsomesal process or spine (e.g., *Itaura amazonica*). *Mortoniella* has a similar dorsomesal process or spine, but in *Mortoniella* it arises internally from the phallobase, whereas in *Itauara* it arises dorsobasally, as an extension of the phallicata. In several species the sheath produces a dorsolateral flange-like process, although this character is not diagnostic for the genus. Another genitalic feature characteristic of *Itauara* is an extremely reduced phallobase. In most species, the phallobase is barely visible, consisting of a small, very lightly sclerotized or an entirely membranous structure. The genera *Mastigoptila* and *Canoptila* display similar reductions or absences of the phallobase, but can easily be separated from *Itauara* by other genitalic characters: *Mastigoptila* has an elongate, whip-like process arising from the membranes of the phallocrypt; *Canoptila* has highly membranous digitate parameres. When present (they have been lost in many species), the inferior appendages are rather distinct for *Itauara*, consisting of a single or apically bifid process produced mesally and fused to the phallobase ventrobasally. This inferior appendage process articulates with the base of the phallobase and in doing so, is capable of pivoting downward (Fig. 13A and inset). All species, except in *Itaura brasiliana*, have rather elongate, sclerotized, rod-like parameres, whose shape varies greatly among species. In many species these parameres arise ventrobasally from the phallobase, with which they appear to articulate. As the inferior appendage process is absent in those species, it is possible that the parameres have taken on a clasper-like function.

**Figure 2.**
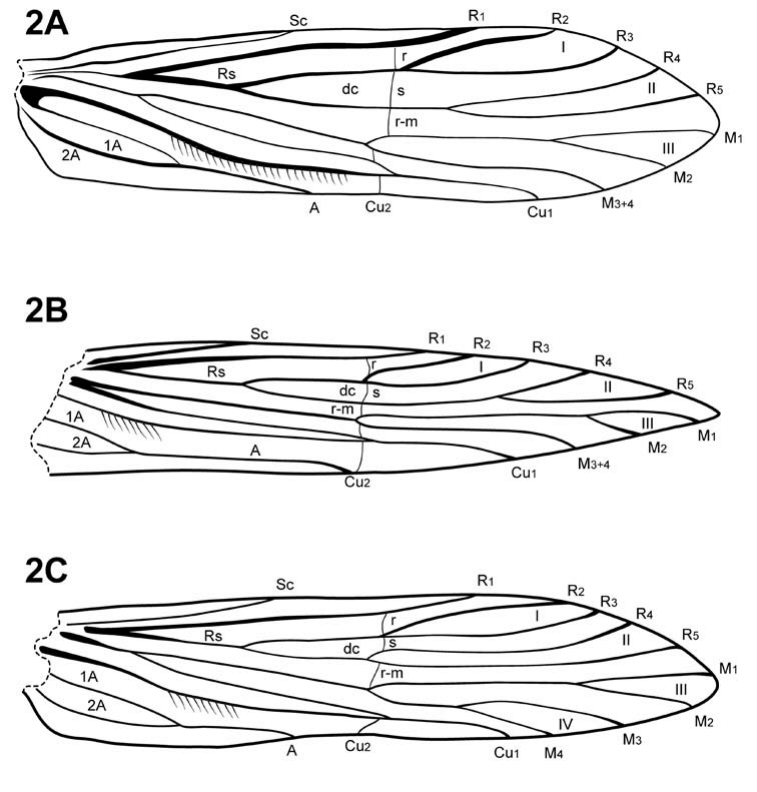
Forewings. (**A**) *Itauara brasiliana* (Mosely). (**B**) *Itauara guyanensis* sp. n. (**C**) *Itauara unidentata* sp. n. Wings between taxa not to scale.

**Figure 3.**
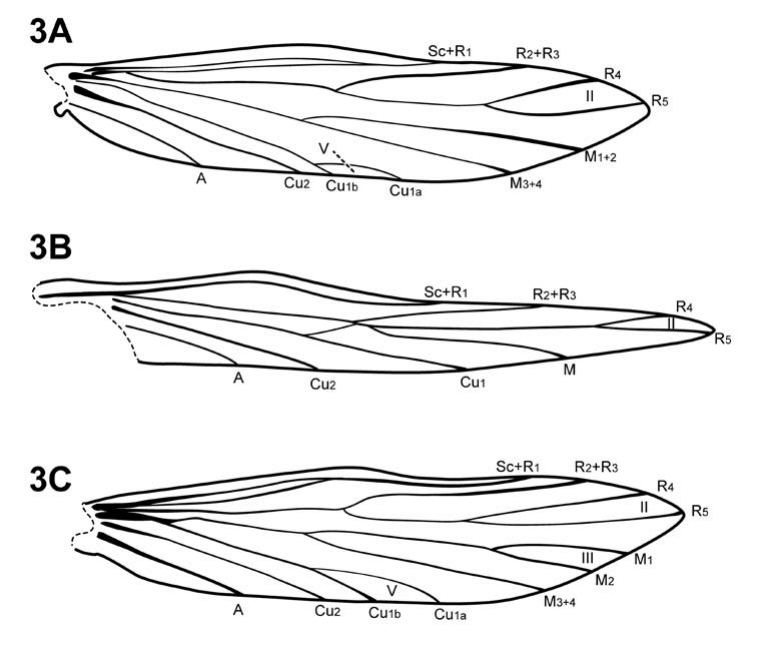
Hind wings. (**A**) *Itauara brasiliana* (Mosely). (**B**) *Itauara guyanensis* sp. n. (**C**) *Itauara julia* sp. n. Wings between taxa not to scale.

The forewing venation of *Itauara* is most similar to that of *Cariboptila* and *Canoptila*, with apical forks I–III and a lack of 3A (Fig. 2A, B). A single species also possesses apical fork IV (Fig. 2C). *Canoptila* can be differentiated from *Itauara* by having stout setae occurring below Cu2 whereas in *Itauara* the setae occur along the vein. *Cariboptila* can be differentiated from *Itauara* by the presence of a short discoidal cell, that of *Itauara* being long. The lengths of the apical forks vary among species. The hind wing venation of *Itauara* is variable, with either apical forks II, III, and V (Fig. 3C); II and V (Fig. 3A); III only, or II only (Fig. 3B).

Adult. Body, wings, and appendages pale or tawny brown, often intermingled with rufous or golden hairs, tibia and tarsi yellowish brown (Fig. 1). Wings often with partial white transverse line along anastomosis not reaching costal margin, or often with conspicuous white spot at the arculus (Fig. 1). Head broader than long, vertex rounded, with pair of small anteromesal setal warts or with large anteromesal setal wart, either 1 distinct pair or 1 divided pair of suboval anterior setal warts, small or large suboval posterior warts, suboval or triangular and bulging posterolateral setal warts. Ocelli present. Antennal scape less than or equal to 2 times the length of pedicel. Maxillary palps 5 segmented, 1st and 2nd segments short; 2nd segment bulbous; last 3 segments each nearly same length as 1st and 2nd segments combined. Prothorax with 2 large subtriangular or suboval pronotal setal warts. Mesothorax wider than long, without apparent tegular glands; mesoscutum with pair of suboval anteromesal setal warts, suboval posterolateral warts; mesoscutellum sparsely setose, without distinct setal warts. Forewing (Fig. 2) usually relatively narrow, with margins nearly parallel, occasionally narrowed past anastomosis or much reduced, apex acute, subacute, or rounded. Male occasionally with callosity present in apical costal region of forewing. Forewing venation incomplete, with apical forks I, II, and III present, or rarely I–IV present; Sc and R1 distinct along their entire lengths; fork I sessile or only slightly petiolate with extremely short stem; fork II petiolate or sessile, when petiolate, stem length variable; fork III petiolate, stem variable in length; Cu1 complete, reaching wing margin; Cu1 and Cu2 intersecting near anastomosis; row of erect setae present along Cu2; A3 absent; crossveins forming a relatively linear transverse cord; discoidal cell longer than Rs vein. Hind wing (Fig. 3) margins nearly parallel, tapering only slightly past anastomosis, or narrowed, scalloped past anastomosis, or much reduced; venation variable, either with apical forks II, III, and V present, II and V present, III present, or II present; Sc and R1 fused basally or converging near wing margin; A2 absent. Tibial spurs 1,4,4, rarely 1,3,4, foretibial spur extremely reduced and hairlike. Sixth sternal process present, short and digitate or thumb-like and prominent, apex rounded or attenuate and pointed, usually associated with oblique apodeme posteriorly.

Male genitalia. Segment IX usually rather broad, anterior margin rounded, posterolateral margin without lateral process or lobes in lateral view; tergum IX usually not well developed, simple, and without processes; sternum IX without modification, except in *Itaura brasiliana*, which bears 2 pairs of elongate, seta-like processes. Tergum X incompletely fused to tergum IX ventrolaterally or rarely (*Itaura amazonica*) completely fused and indistinct from tergum IX, shape extremely variable; dorsomesal margin may be simple without processes, bifid apicomesally, with a single broad, plate-like process, or irregular with several small processes; dorsolateral margin either a simple structure without processes, or more commonly with small paired lobes, elongate, down-turned, finger-like process, or irregular setose processes; ventrolateral margin with paired elongate or broad flange-like processes directed ventrally and sometimes anteriorly, or with one or more irregular, paired, setose, digitate lobes directed posteriorly. Inferior appendages either present or absent; when present, consisting of single or apically bifid process produced mesally, broadest at base and fused to phallobase ventrobasally. Parameres present except in *Itaura brasiliana*, arising either ventrobasally from phallobase or laterally from endotheca, sclerotized, shape variable. Phallobase extremely reduced and difficult to discern. Phallicata a sclerotized dorsal sheath covering membranous ventral portion, sometimes receding to a single dorsomesal process arising dorsobasally from phallobase, phallicata occasionally with dorsolateral flange, or occasionally with dorsomesal spine arising posteriorly to phallobase. Endophallus highly membranous, enlarged and convoluted when evaginated, occasionally bearing apical spine-like sclerites and processes.

Female genitalia. (Females unknown for many species.) Truncate posteriorly, not extensible. Abdominal segment VIII short, synscleritous, posterolateral margin slightly incised. Segments IX and X closely associated, with pair of small digitate cerci dorsolaterally.

## Species relationships

The 22 species of *Itauara* fall into 2 broad species groups (Table 1). Members of the *amazonica* species group are recognized by the presence of a fused inferior appendage process. The group is also characterized by the position of the parameres, arising laterally from the endotheca in this group. Species included in the *brasiliana* species group have completely lost the inferior appendages. When parameres are present (they are vestigial in *Itaura brasiliana*) they arise ventrobasally from the phallobase, to which they are often fused. Several members of the *brasiliana* group also have lateral flange-like processes on the phallicata.

## Species descriptions

### Itauara alexanderi

Robertson & Holzenthal sp. n.

urn:lsid:zoobank.org:act:55610B1E-3E37-4B64-9380-5C2A86CD3EA1

http://species-id.net/wiki/Itauara_alexanderi

Fig. 4A–C 

#### Description.

 This species is similar to *Itauara emilia* sp. n., *Itaura lucinda* sp. n., and *Itaura stella* sp. n., as discussed under each of those species. Each of these species possesses an inferior appendage process, a dorsomesal process on tergum X, and rather sinuous parameres. Of these species, *Itaura alexanderi* is most similar to *Itaura stella*. Both of these species have similarly shaped dorsomesal and ventrolateral processes of tergum X and both have apically bifid inferior appendage processes. *Itauara alexanderi* can be distinguished from *Itaura stella* by the length of the parameres, those of *Itaura alexanderi* being much shorter. Additionally, the inferior appendage process of *Itaura alexanderi* is broader than that of *Itaura stella*. *Itauara lucinda* differs from *Itaura alexanderi* in having a forked paramere and an inferior appendage process that is not bifid. *Itauara alexanderi* can be distinguished from *Itaura emilia* based on differences in the shape of the dorsomesal process of tergum X.

Adult. Body, wings, and appendages fuscous, intermingled with rufous or golden hairs, tibia and tarsi tawny brown. Wings with white transverse line along anastomosis. Forewing slightly broader past anastomosis, but with margins nearly parallel, apex rounded. Forewing venation incomplete, with apical forks I, II, and III present; Sc and R1 distinct along their entire lengths; fork I sessile; fork II petiolate, stem about the same length as fork; fork III petiolate, stem longer than fork; Cu1 complete, reaching wing margin; Cu1 and Cu2 intersecting near anastomosis; row of erect setae present along Cu2; A3 absent; crossveins forming a relatively linear transverse cord; discoidal cell longer than Rs vein. Hind wing margins nearly parallel, tapering only slightly past anastomosis; apical forks II and V present; Sc and R1 fused basally; A2 absent. Tibial spurs 1,4,4, foretibial spur extremely reduced and hairlike. Sixth sternal process thumb-like, apex rounded, associated with oblique apodeme posteriorly.

Male genitalia. Preanal appendages absent. Segment IX ventrally narrow, broad medially; anterior margin rounded; posterolateral margin membranous or very lightly sclerotized; sternum IX without modification. Tergum X incompletely fused to tergum IX with membrane or lightly sclerotized region ventrolaterally; dorsomesal margin with single, downturned, elongate process; dorsolateral margin without processes; ventrolateral margin with paired, broad flange-like setose process consisting of upper subtriangular lobe and lower subquadrate lobe. Inferior appendages present as apically bifid, setose process produced mesally, broadest at base and fused to phallobase ventrobasally, with 2 pairs of small digitate lobes ventrolaterally, each bearing a seta. Parameres present, paired, inserted in membranous lobe, arising laterally from endotheca, sclerotized and rod-like, relatively short, sinuous, directed ventrolaterally, apex pointed. Phallobase reduced, lightly sclerotized. Phallicata forming a long sclerotized dorsal sheath extending from phallobase, narrow and straight mesally, distal portion broad, curving dorsally. Endophallus membranous, enlarged and convoluted when invaginated, with 1 upper and 1 lower lobe.

**Figure 4.**
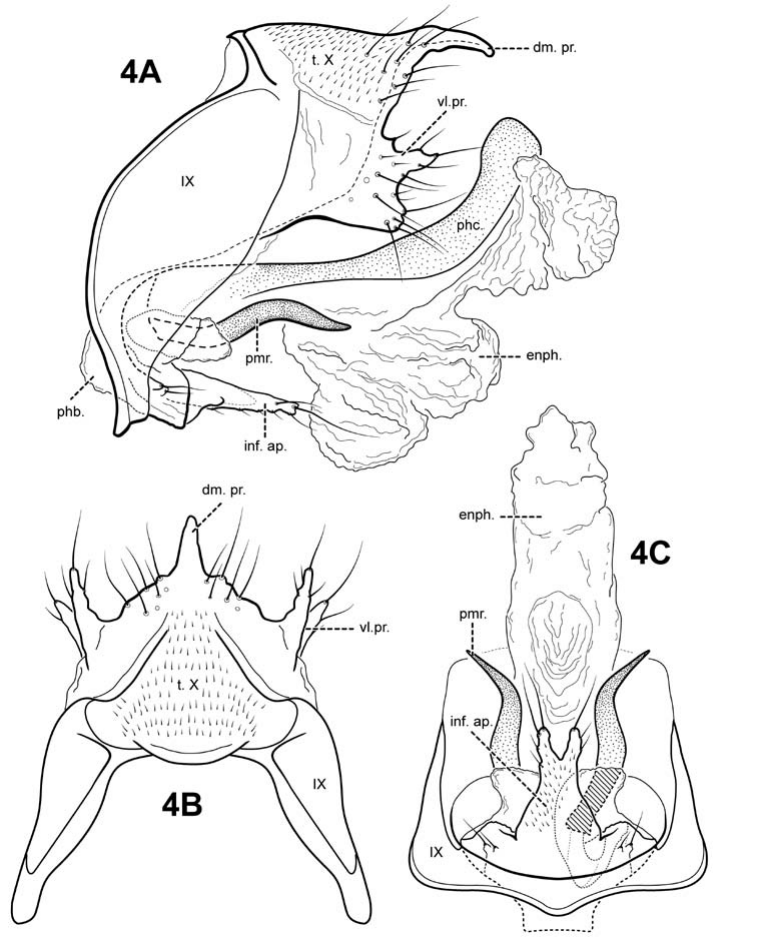
*Itauara alexanderi* sp. n. (composite of UMSP000114626 & UMSP000052590). Male genitalia: (**A**) lateral; (**B**) dorsal; (**C**) ventral. Abbreviations: dm. pr. = dorsomesal process; enph. = endophallus; inf. ap. = inferior appendage process; phb = phallobase; phc. = phallicata; pmr . = paramere; t. X = tergum X; vl. pr. = ventrolateral process.

#### Material examined.

**Holotype male:** **BRAZIL: Nova Friburgo**, 22°16'00"S, 042°31'59"W, 950 m, 20.iv.1977 (C. & O. Flint) (UMSP000052592) (NMNH)

**Paratypes: BRAZIL: Rio de Janeiro**, Teresopolis, 18 km S, Km 17 (road), 1180 m, 18–19.iv.1977 (C. & O. Flint) — 2 males (NMNH).

#### Etymology.

We are delighted to name this species for the senior author’s husband, Alexander Bishop Thompson, in gratitude of his patience, support, and encouragement as she worked to finish her dissertation.

### Itauara amazonica

(Flint, 1971)

http://species-id.net/wiki/Itauara_amazonica

Fig. 5A–D

amazonica  (Flint), 1971:13 [Type locality: Brazil [Edo. Amazonas], Rio Marauiá, Endstation langer Cachoeira, Fluß tritt hier aus dem Gebirge mit starkem Gefálle; NMNH; in *Antoptila*]. –Flint, Holzenthal, and Harris, 1999:74 [to *Itauara*].

#### Description.

 This species is distinct in having a very elongate inferior appendage process, a rather simple tergum X, and sharply bent apical spines in the endophallus. *Itauara amazonica* is most similar to 3 species from Guyana, *Itaura bidentata* sp. n., *Itaura spiralis* sp. n., and *Itaura unidentata* sp. n. These species, including *Itaura amazonica*, all have a dorsomesal spine arising from the phallicata. However, in *Itaura amazonica*, this spine appears as a short, posterior extension of the phallicata, whereas in the other species, the spine arises basally, as a separate spine. *Itaura amazonica* can further be distinguished from these species based on differences in the shape of tergum X and parameres.

Adult. Body, wings, and appendages pale or tawny brown in alcohol. Forewing relatively narrow, with margins nearly parallel, apex subacute. Forewing venation incomplete, with apical forks I, II, and III present; Sc and R1 distinct along their entire lengths; fork I sessile; fork II petiolate, stem shorter than fork; fork III petiolate, stem longer than fork; Cu1 complete, reaching wing margin; Cu1 and Cu2 intersecting near anastomosis; row of erect setae present along Cu2; A3 absent; crossveins forming a relatively linear transverse cord; discoidal cell longer than Rs vein. Hind wing narrow and slightly scalloped past anastomosis; apical fork III present; Sc and R1 fused basally; A2 absent. Tibial spurs 1,4,4, foretibial spur extremely reduced and hairlike. Sixth sternal process short and digitate, apex attenuate and pointed, associated with weak oblique apodeme posteriorly.

Male genitalia. Preanal appendages absent. Segment IX relatively broad; anterior margin rounded; posterolateral margin membranous or very lightly sclerotized; sternum IX without modification. Tergum X completely fused to tergum IX, divided or bifid apicomesally, each half terminating in pointed process directed posteriorly; dorsolateral margin without processes; ventrolateral margin without processes. Inferior appendages present as single, elongate setose process produced mesally, broadest at base and fused to phallobase ventrobasally. Parameres present, paired, inserted in membranous lobe, arising laterally from endotheca, sclerotized and rod-like, slender and elongate, upturned, directed dorsally, apex pointed. Phallobase reduced, lightly sclerotized. Phallicata forming a long sclerotized dorsal sheath extending from phallobase, bent sharply upward at middle, divided apicomesally, terminating in 2 pointed processes. Endophallus membranous, enlarged and convoluted when invaginated, receding anterior to apex of phallicata, ventrally bearing a pair of broad, tooth-like downturned processes, apically with pair of sharply bent sclerotized spines, pointing anteriorly.

**Figure 5.**
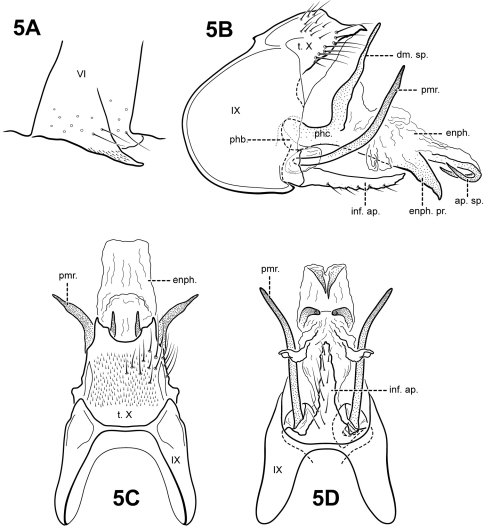
*Itauara amazonica* (Flint, 1971) (UMSP000027156). (**A**) Process of sternum VI. Male genitalia: (**B**) lateral; (**C**) dorsal; (**D**) ventral. Abbreviations: ap. sp. = apical spine; dm. sp. = dorsomesal spine; enph. = endophallus; enph. pr. = endophallic process; inf. ap. = inferior appendage process; phb = phallobase; phc. = phallicata; pmr . = paramere; t. X = tergum X.

#### Material examined.

**Holotype male:** **BRAZIL: Amazonas:** Rio Marauia, Endstation langer Cachoeira, Fluß tritt hier aus dem Gebirge mit starkem Gefälle, 00°23'00"N, 065°13'00"W, 28.i.1963 (E.J. Fittkau) (UMSP000027159) (NMNH).

**Paratypes: BRAZIL: Amazonas:** same data as holotype — 3 males, 2 females (NMNH).

### Itauara bidentata

Robertson & Holzenthal sp. n.

urn:lsid:zoobank.org:act:76A03637-7788-4644-975B-4AC7F58E37EA

http://species-id.net/wiki/Itauara_bidentata

Fig. 6A–C 

#### Description.

 *Itauara bidentata* can be diagnosed by its large, bifid paramere process, and spade-like shaped inferior appendage. It is most similar to *Itaura unidentata* sp. n., which has a similarly shaped tergum X, dorsomesal spine, and apical sclerites. The 2 species can be separated by their paramere processes; in *Itaura unidentata* the paramere consists of a single large tooth-like spine, whereas in *Itaura bidentata*, the paramere process is bifid. *Itauara spiralis* sp. n., has a similarly shaped tergum X, but is easily distinguished from *Itaura bidentata* by differences in the shape of the inferior appendage process, parameres, and phallicata.

Adult. Body, wings, and appendages pale or tawny brown in alcohol. Forewing relatively narrow, with margins nearly parallel, apex subacute. Forewing venation incomplete, with apical forks I, II, and III present; fork I sessile; fork II petiolate, stem about the same length as fork; fork III petiolate, stem longer than fork; Cu1 complete, reaching wing margin; Cu1 and Cu2 intersecting near anastomosis; row of erect setae present along Cu2; A3 absent; crossveins forming a relatively linear transverse cord; discoidal cell longer than Rs vein. Hind wing narrow and slightly scalloped past anastomosis; apical fork II present; Sc and R1 fused basally; A2 absent. Tibial spurs 1,4,4, foretibial spur extremely reduced and hairlike. Sixth sternal process short and digitate, apex attenuate and pointed, associated with strong oblique apodeme posteriorly.

Male genitalia. Preanal appendages absent. Segment IX ventrally narrow, broad medially; anterior margin rounded; posterolateral margin membranous or very lightly sclerotized; sternum IX without modification. Tergum X incompletely fused to tergum IX with membrane or lightly sclerotized region ventrolaterally; dorsomesal margin straight, without processes; dorsolateral margin with paired elongate, down-turned, finger-like process; ventrolateral margin with paired, broad flange-like setose process consisting of several small irregular lobes. Inferior appendages present as single, broad, irregular setose process, broadest basally, fused to phallobase ventrobasally, bearing a single pair of small digitate lobes ventrolaterally, each bearing a seta. Parameres present, paired, arising laterally from endotheca, strongly sclerotized, large bifid tooth-like process, curving ventrally and outward, apices pointed. Phallobase reduced, lightly sclerotized dorsally, laterally membranous, with 2 irregular and elongate sclerites arising basolaterally. Phallicata forming a short slerotized dorsal sheath with an elongate dorsomesal spine arising posteriorly to phallobase. Endophallus membranous, enlarged and convoluted when invaginated, apically bearing 3 small sclerotized spines.

**Figure 6.**
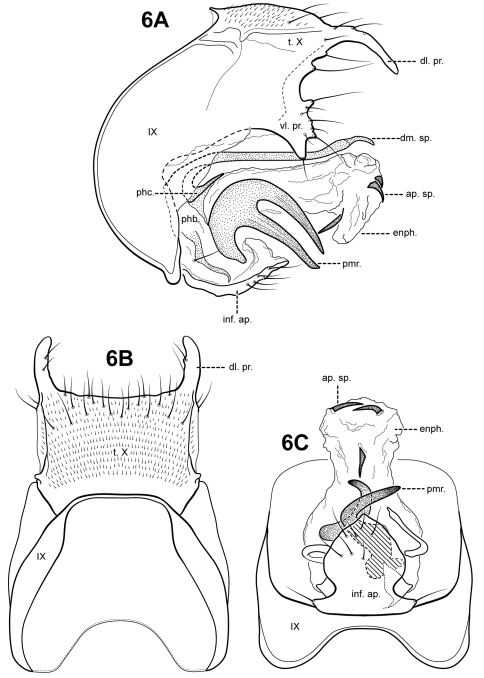
*Itauara bidentata* sp. n. (UMSP000210958). Male genitalia (**A**) lateral; (**B**) dorsal; (**C**) ventral. Abbreviations: ap. sp. = apical spine; dl. pr. = dorsolateral process; dm. sp. = dorsomesal spine; enph. = endophallus; inf. ap. = inferior appendage process; phb. = phallobase; phc. = phallicata; pmr . = paramere; t. X = tergum X; vl. pr. = ventrolateral process.

#### Material examined.

**Holotype male: GUYANA: KUMU:** 25 km. SE Lethem, 03°15'54"N, 059°43'36"W, 4–5.iv.1994 (O.S. Flint) (UMSP000127385) (NMNH)

**Paratypes: GUYANA: Kumu**: same data as holotype — 1 male, 3 females.

#### Etymology.

The name *bidentata* comes from the bidentate form of the paramere process.

### Itauara blahniki

Robertson & Holzenthal sp. n.

urn:lsid:zoobank.org:act:2ED84A59-BC4E-4140-843D-DC11FFE4B8E6

http://species-id.net/wiki/Itauara_blahniki

Fig. 7A–C 

#### Description.

 *Itauara blahniki* can be recognized by the shape of the phallicata, which has a pair of very short spines dorsobasally, and a sclerotized lobe ventrobasally. The ventrolateral process of tergum X is also distinct, consisting of a an outwardly projecting flange-like setose process, and an inner, small digitate setose process. *Itauara blahniki* has elongate, tusk-like parameres. *Itauara rodmani* sp. n., and *Itaura tusci* sp. n., also have tusk-like parameres, but unlike *Itaura blahniki*, these species have flange-like lateral processes on the phallicata. The species also have differently shaped terga X.

Adult. Body, wings, and appendages pale or tawny brown, often intermingled with rufous or golden hairs, tibia and tarsi tawny brown. Wings with conspicuous white spot at the arculus. Forewing slightly broader past anastomosis, but with margins nearly parallel, apex rounded. Forewing venation incomplete, with apical forks I, II, and III present; Sc and R1 distinct along their entire lengths; fork I sessile; fork II sessile; fork III petiolate, stem about the same length as fork; Cu1 complete, reaching wing margin; Cu1 and Cu2 intersecting near anastomosis; row of erect setae present along Cu2; A3 absent; crossveins forming a relatively linear transverse cord; discoidal cell longer than Rs vein. Hind wing margins nearly parallel, tapering only slightly past anastomosis; apical forks II, III, and V present; Sc and R1 fused basally; A2 absent. Tibial spurs 1,4,4, foretibial spur extremely reduced and hairlike. Sixth sternal process thumb-like, apex rounded, associated with oblique apodeme posteriorly.

Male genitalia. Preanal and inferior appendages absent. Segment IX dorsally and ventrally narrow, broad medially; anterior margin rounded; posterolateral margin membranous or very lightly sclerotized; sternum IX without modification. Tergum X incompletely fused to tergum IX with membrane or lightly sclerotized region ventrolaterally; dorsomesal margin with single, downturned, elongate process; dorsolateral margin irregular and setose; ventrolateral margin with paired, outwardly projecting flange-like setose process, and medially with paired digitate setose process. Parameres present, paired, arising ventrobasally from fused endotheca and phallobase, sclerotized and rod-like, slender and elongate, upturned, with distal portion slightly broader, directed dorsally, apex pointed, ventrobasally with small patch of setae. Phallobase reduced, mostly membranous, ventromesally bearing pair of small sclerotized spines. Phallicata forming a long sclerotized dorsal sheath, curving upward, dorsobasally with pair of short processes, ventrally, with lightly sclerotized lobe. Endophallus membranous, enlarged and convoluted when invaginated, with lightly sclerotized lobe ventrally.

#### Material examined.

 **Holotype male: BRAZIL: Sao Paulo**: Estação Biológica Boraceia, Rio Guaratuba, 23°40'02"S, 045°53'46"W, 775 m, 17.ix.2002 (Blahnik, Prather, Melo, Froehlich, Silva) (UMSP000087057) (MZUSP).

**Paratypes: BRAZIL: Sao Paulo:** same data as holotype except 17.iv.1998 (Holzenthal, Melo, Froehlich) — 1 male (UMSP); same data as holotype — 1 female (UMSP).

**Figure 7.**
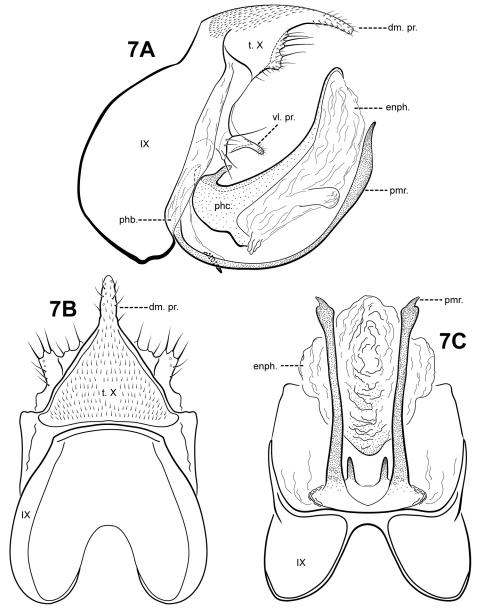
*Itauara blahniki* sp. n. (UMSP000087057) Male genitalia: (**A**) lateral; (**B**) dorsal; (**C**) ventral. Abbreviations: dm. pr. = dorsomesal process; enph. = endophallus; phb = phallobase; phc. = phallicata; pmr . = paramere; t. X = tergum X; vl. pr. = ventrolateral process.

#### Etymology.

We are delighted to name this species for Dr. Roger Blahnik, in honor of his many contributions to the systematics of Protoptilinae.

### Itauara brasiliana

(Mosely 1939)

http://species-id.net/wiki/Itauara_brasiliana

Fig. 2A 3A 8A–C

brasiliana  (Mosely), 1939: 220 [Type locality: Brazil, Santa Catarina, Nova Teutonia; BMNH] in *Antoptila*]. –Angrisano, 1993: 59 [larva, pupa, case, distribution] 1997:58 [distribution] – Flint, Holzenthal, and Harris, 1999:74 [to *Itauara*].

#### Description.

 This species is easily diagnosed by the presence of 2 pairs of extremely elongate, seta-like processes on sternum IX. *Itauara brasiliana* is also distinct in having vestigial parameres, consisting only of very small, setose lobes. Another distinguishing characteristic is the shape of segment IX, which is rather narrow, and receded ventrally. The rather elongate profile of tergum X, as well as the shape of the dorsomesal and lateral margins of tergum X, slightly resembles that of *Itaura plaumanni* (Flint 1974). However, *Itaura plaumanni* has much more pronounced, elongate parameres, and lacks the seta-like processes on sternum IX.

Adult. Body, wings, and appendages pale or tawny brown in alcohol. Forewing slightly broader past anastomosis, but with margins nearly parallel, apex rounded. Forewing venation incomplete, with apical forks I, II, and III present; Sc and R1 distinct along their entire lengths; fork I sessile; fork II petiolate, stem shorter than fork; fork III petiolate, stem about the same length as fork; Cu1 complete, reaching wing margin; Cu1 and Cu2 intersecting near anastomosis; row of erect setae present along Cu2; A3 absent; crossveins forming a relatively linear transverse cord; discoidal cell longer than Rs vein. Hind wing margins nearly parallel, tapering only slightly past anastomosis; apical forks II and V present; Sc and R1 converging near wing margin; A2 absent. Tibial spurs 1,4,4, foretibial spur extremely reduced and hairlike. Sixth sternal process thumb-like, apex rounded, associated with oblique apodeme posteriorly.

Male genitalia. Preanal and inferior appendages absent. Segment IX ventrally narrow, broad medially; anterior margin relatively straight from dorsum to medial area, ventral portion rounded; posterolateral margin highly membranous, receding ventrally; sternum IX bearing 2 pairs of extremely elongate, seta-like processes. Tergum X incompletely fused to tergum IX with membrane or lightly sclerotized region ventrolaterally; dorsomesal margin subtriangular, slightly upturned; dorsolateral margin slightly irregular, without processes; ventrolateral margin with 2 pairs of processes, the upper an elongate finger-like process slightly downturned, the lower a smaller lobe-like setose process. Parameres vestigial, consisting of a pair of small, digitate setose lobes arising ventrolaterally from endotheca. Phallobase apparently absent or entirely membranous. Phallicata forming a long, lightly sclerotized dorsal sheath, sinuous, broadest medially, narrowed distally. Endophallus membranous, enlarged and convoluted when invaginated.

**Figure 8.**
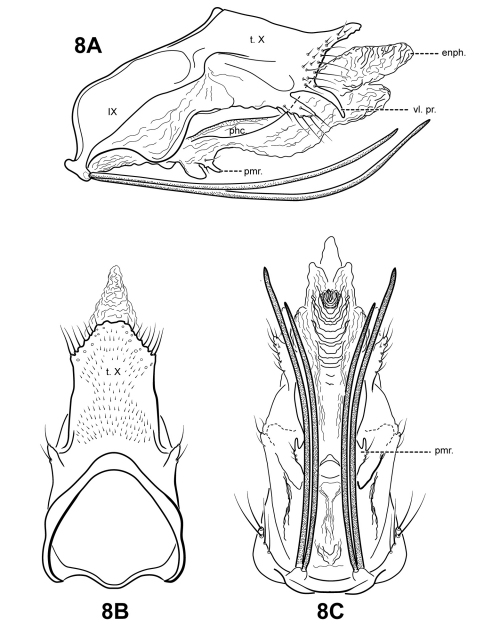
*Itauara brasiliana* (Mosely, 1939). Male genitalia. (**A**) lateral; (**B**) dorsal; (**C**) ventral. Abbreviations: enph. = endophallus; phc. = phallicata; pmr . = paramere; t. X = tergum X; vl. pr. = ventrolateral process.

#### Material examined.

**Holotype male: Brazil: Santa Catarina**: Nova Teutonia, ii. 1937 (F. Plaumann) (BMNH)

#### Additional material examined:

 **ARGENTINA: Misiones:** Arroyo Piray Mini, W., Dos Hermanas, 23.11.1973 (O.S. Flint) — 5 males, 14 females (NMNH); **BRAZIL: Santa Catarina:** Seara (Nova Teutônia), 27°11'00"S, 052°23'00"W, 300–500 m, 10.1964 (F. Plaumann) — 1 male (NMNH).

### Itauara charlotta

Robertson & Holzenthal sp. n.

urn:lsid:zoobank.org:act:EE8E2255-6A12-407B-9D1F-921D251401B7

http://species-id.net/wiki/Itauara_charlotta

Fig. 9A–C 

#### Description.

 This species is only known from the male holotype. *Itauara charlotta* is diagnosed by the unique combination of several male genitalic characters. The dorsomesal margin of tergum X has several small, irregular, setose processes. *Itauara tusci* sp. n., has a similarly shaped dorsomesal margin, but differs in the shape of the parameres and phallicata. The parameres of *Itaura charlotta* are similar to those of *Itaura flinti* sp. n.; both arise ventrobasally from the phallobase, and are curved and downturned. *Itauara flinti* is easily separated from *Itaura charlotta* based on differences in the shape of tergum X and phallicata processes.

Adult. Body, wings, and appendages pale or tawny brown in alcohol. Wings with conspicuous white spot at the arculus and faint transverse line along anastomosis. Forewing slightly broader past anastomosis, but with margins nearly parallel, apex rounded. Forewing venation incomplete, with apical forks I, II, and III present; Sc and R1 distinct along their entire lengths; fork I sessile; fork II petiolate, stem shorter than fork; fork III petiolate, stem longer than fork; Cu1 complete, reaching wing margin; Cu1 and Cu2 intersecting near anastomosis; row of erect setae present along Cu2; A3 absent; crossveins forming a relatively linear transverse cord; discoidal cell longer than Rs vein. Hind wing margins nearly parallel, tapering only slightly past anastomosis; apical forks II, III, and V present; Sc and R1 fused basally; A2 absent. Tibial spurs 1,4,4, foretibial spur extremely reduced and hairlike. Sixth sternal process thumb-like, apex rounded, often associated with weak oblique apodeme posteriorly.

Male genitalia. Preanal and inferior appendages absent. Segment IX ventrally narrow, broad medially; anterior margin rounded; posterolateral margin membranous or very lightly sclerotized; sternum IX without modification. Tergum X incompletely fused to tergum IX with membrane or lightly sclerotized region ventrolaterally; dorsomesal margin slightly produced with several small irregular setose processes; dorsolateral margin with pair of large irregular, setose process and several smaller processes; ventrolateral margin without processes. Parameres present, paired, arising ventrobasally from fused endotheca and phallobase, sclerotized and rod-like, slender and elongate, slightly downturned, curved basally, straight medially and distally, directed posteriorly, apex pointed. Phallobase reduced, lightly sclerotized. Phallicata forming a long, lightly sclerotized dorsal sheath, slightly sinuous, medially with pair of slender lateral flanges projecting posteroventrally, apices pointed. Endophallus membranous, enlarged and convoluted when evaginated, with pointed apical sclerite.

**Figure 9.**
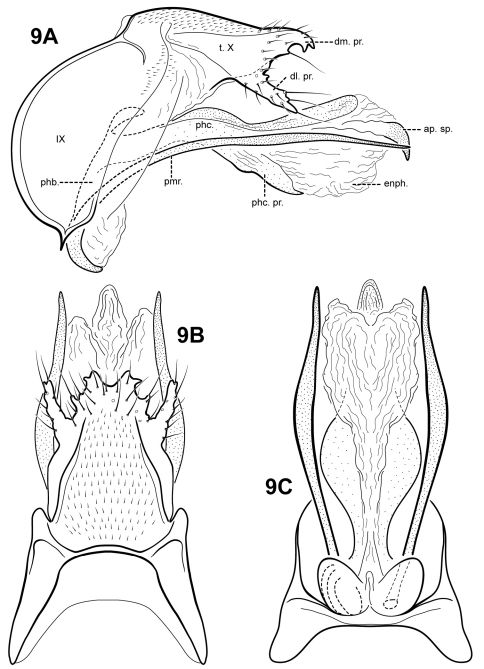
*Itauara charlotta*, sp. n. (UMSP000086390). Male genitalia: (**A**) lateral; (**B**) dorsal; (**C**) ventral. Abbreviations: ap. sp. = apical spine; dl. pr. = dorsolateral process; dm. pr. = dorsomesal process; enph. = endophallus; phb = phallobase; phc. = phallicata; phc. pr. = phallicata process; pmr . = paramere; t. X = tergum X.

#### Material examined.

 **Holotype male: BRAZIL: Minas Gerais:** Serra do Cipó, Cardeal Mota, Cachoeira Veu da Noiva, 19°18'55"S, 043°36'16"W, 800 m, 12.11.2001 (Holzenthal, Amar., Blahnik, Paprocki) (UMSP000086390) (MZUSP).

#### Etymology.

We are delighted to name this species for the senior author’s mother, Charlotte Ruth Robertson.

### Itauara emilia

Robertson & Holzenthal sp. n.

urn:lsid:zoobank.org:act:EDA5DA9E-6661-4F15-96A2-B6166861CA49

http://species-id.net/wiki/Itauara_emilia

Fig. 10A–C 

#### Description.

 This species is known only from the male holotype. *Itauara emilia* can be recognized by the distinct, rather blunt shape of the dorsomesal process of tergum X. The species is similar to *Itaura alexanderi* sp. n., *Itaura lucinda* sp. n., and *Itaura stella* sp. n., as discussed under each of those species. Each of these species possess an inferior appendage process, a dorsomesal process on tergum X, and rather sinuous parameres. *Itauara emilia* is most similar to *Itaura alexanderi* and *Itaura stella* in having similarly shaped parameres and an apically bifid inferior appendage process. *Itauara emilia* differs from these 2 species in having a much more elongate inferior appendage process and a blunt dorsomesal process on tergum X.

Adult. Body, wings, and appendages tawny brown (specimen missing hairs). Forewing slightly broader past anastomosis, but with margins nearly parallel, apex rounded. Forewing venation incomplete, with apical forks I, II, and III present; Sc and R1 distinct along their entire lengths; fork I sessile; fork II petiolate, stem about the same length as fork; fork III petiolate, stem longer than fork; Cu1 complete, reaching wing margin; Cu1 and Cu2 intersecting near anastomosis; row of erect setae present along Cu2; A3 absent; crossveins forming a relatively linear transverse cord; discoidal cell longer than Rs vein. Hind wing margins nearly parallel, tapering only slightly past anastomosis; apical forks II and V present; Sc and R1 fused basally; A2 absent. Tibial spurs 1,4,4, foretibial spur extremely reduced and hairlike. Sixth sternal process thumb-like, apex rounded, associated with oblique apodeme posteriorly.

Male genitalia. Preanal appendages absent. Segment IX ventrally narrow, broad medially; anterior margin rounded; posterolateral margin membranous or very lightly sclerotized; sternum IX without modification. Tergum X incompletely fused to tergum IX with membrane or lightly sclerotized region ventrolaterally; dorsomesal margin with large, blunt, dorsomesal process, in dorsal view, elongate, in lateral view, subtriangular; dorsolateral margin without processes; ventrolateral margin with paired, broad flange-like setose process consisting of small upper lobe and larger subtriangular lower lobe. Inferior appendages present as apically bifid, setose process produced mesally, broadest at base and fused to phallobase ventrobasally, with 2 pairs of small digitate lobes ventrolaterally, each bearing a seta. Parameres present, paired, inserted in membranous lobe, arising laterally from endotheca, sclerotized and rod-like, long, sinuous, directed inward and posteriorly, apex pointed. Phallobase reduced, lightly sclerotized. Phallicata forming a long sclerotized dorsal sheath extending from phallobase, straight, broadest basally, distal portion narrow. Endophallus membranous, enlarged and convoluted when invaginated, with 1 upper and 1 lower lobe.

**Figure 10.**
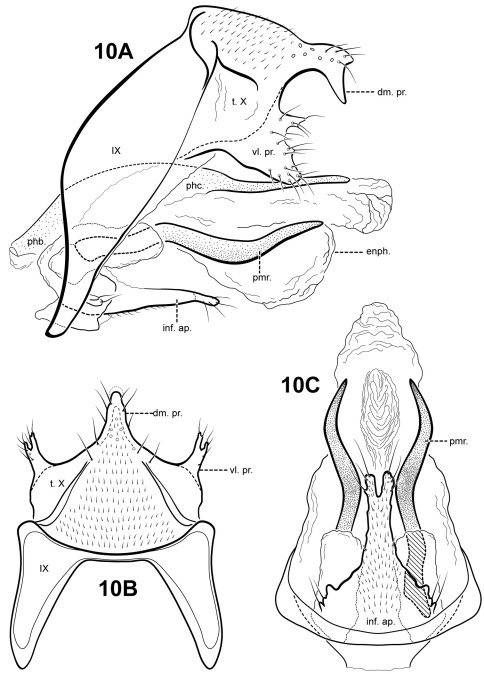
*Itauara emilia* (UMSP000029788). Male genitalia (**A**) lateral; (**B**) dorsal; (**C**) ventral. Abbreviations: dm. pr. = dorsomesal process; enph. = endophallus; inf. ap. = inferior appendage process; phb. = phallobase; phc. = phallicata; pmr . = paramere; t. X = tergum X; vl. pr. = ventrolateral process.

#### Material examined.

 **Holotype male: BRAZIL: Sao Paulo:** Estação Biológica Boraceia, Rio Coruja, 23°40'06"S, 045°53'57"W, 850 m, 18.iv.1998 (Holzenthal, Melo, Froehlich) (UMSP000029788) (MZUSP).

#### Etymology.

This species is named in loving memory of the senior author’s paternal grandmother, Grace Emily Gardner Robertson.

### Itauara flinti

Robertson & Holzenthal sp. n.

urn:lsid:zoobank.org:act:08207511-83D1-46A4-8168-BAE2935AAC0A

http://species-id.net/wiki/Itauara_flinti

Fig. 11A–C 

#### Description.

 This species is known only from the male holotype. It is diagnosed by a unique combination of male genitalic characters and can be recognized by the shape of the phallicata process. *Itauara flinti* is most similar to *Itaura charlotta* sp. n., by having similarly shaped parameres, but these species are easily separated based on differences in the shape of tergum X and the phallicata process. The elongate and downturned dorsomesal process of tergum X is similar to that of *Itaura guarani* (Angrisano 1993), but the 2 species differ in the shape of the parameres and phallicata processes. The ventrolateral process of tergum X is similar to that of *Itaura tusci* sp. n. These species differ in the shape of the parameres, dorsomesal margins of tergum X, and phallicata processes.

Adult. Body, wings, and appendages pale or tawny brown in alcohol. Forewing relatively narrow, with margins nearly parallel, apex subacute. Forewing venation incomplete, with apical forks I, II, and III present; Sc and R1 distinct along their entire lengths; fork I petiolate, but with extremely short stem; fork II petiolate, stem about the same length as fork; fork III petiolate, stem longer than fork; Cu1 complete, reaching wing margin; Cu1 and Cu2 intersecting near anastomosis; row of erect setae present along Cu2; A3 absent; crossveins forming a relatively linear transverse cord; discoidal cell longer than Rs vein. Hind wing narrow and slightly scalloped past anastomosis; apical forks II and V present; Sc and R1 fused basally; A2 absent. Tibial spurs 1,4,4, foretibial spur extremely reduced and hairlike. Sixth sternal process thumb-like, apex rounded, associated with weak oblique apodeme posteriorly.

Male genitalia. Preanal and inferior appendages absent. Segment IX dorsally narrow, broad ventrally; anterior margin rounded; posterolateral margin lightly sclerotized; sternum IX without modification. Tergum X incompletely fused to tergum IX with membrane or lightly sclerotized region ventrolaterally; dorsomesal margin with single, downturned, elongate process; dorsolateral margin irregular and setose; ventrolateral margin with 2 pairs of processes, the upper a small lobe-like setose process, the lower an elongate finger-like process bearing a few elongate setae. Parameres present, paired, arising ventrobasally from fused endotheca and phallobase, sclerotized and rod-like, slender and elongate, downturned, curved basally, straight medially and distally, directed ventrally and inward, apex pointed. Phallobase reduced, lightly sclerotized. Phallicata forming a long, lightly sclerotized dorsal sheath, slightly sinuous, medially with pair of lightly sclerotized rounded lateral flanges projecting posteroventrally, ventrally with several sclerotized points, thorn-like apices directed inward. Endophallus membranous, enlarged and convoluted when invaginated, with 1 large upper lobe and 1 smaller lower lobe.

**Figure 11.**
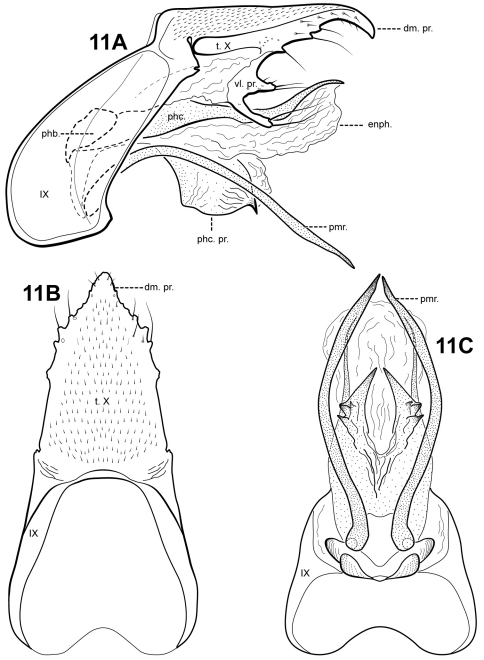
*Itauara flinti* sp. n. (UMSP000086388) Male genitalia: (**A**) lateral; (**B**) dorsal; (**C**) ventral. Abbreviations: dm. pr. = dorsomesal process; enph. = endophallus; phb = phallobase; phc. = phallicata; phc. pr. = phallicata process; pmr . = paramere; t. X = tergum X; vl. pr. = ventrolateral process.

#### Material examined.

**Holotype male: BRAZIL:** **Sao Paulo:** Parque Estadual de Campos do Jordão, Rio Galharada, 22°41'40"S, 045°27'47"W, 1530 m, 13–15.ix.2002 (Blahnik, Prather, Melo, Huamantinco) (UMSP000086388) (MZUSP).

#### Etymology.

We are delighted to name this species for Dr. Oliver Flint, Jr. in honor of his numerous important contributions to Neotropical caddisfly taxonomy.

### Itauara guarani

(Angrisano, 1993)

http://species-id.net/wiki/Itauara_guarani

Fig. 12A–D

guarani  (Angrisano), 1993: 57 [Type locality: Argentina, Misiones, Dpt. Belgrano, Río Urugua-í; MACN; in *Antoptila*] – Flint, Holzenthal, and Harris, 1999:74 [to *Itauara*].

#### Description. 

This species can be recognized by the very broad, lateral flanges on the phallicata. The phallicata of *Itaura guarani* is slightly sinuous, with a lightly sclerotized basal portion and a rugous or almost membranous distal portion. *Itauara simplex* sp. n., also has a very lightly sclerotized phallicata, but the 2 species differ in the shape of the parameres and *Itaura simplex* lacks the processes of phallicata. The parameres of *Itaura guarani* arise ventrobasally from the phallobase and are sinuous, like those in *Itaura plaumanni*. However, the phallicata in *Itaura plaumanni* is much more sclerotized and the 2 species also differ in the shape of tergum X and the phallicata processes.

Adult. Body, wings, and appendages pale or tawny brown in alcohol. Forewing slightly broader past anastomosis, but with margins nearly parallel, apex rounded. Forewing venation incomplete, with apical forks I, II, and III present; Sc and R1 distinct along their entire lengths; fork I petiolate, but with extremely short stem; fork II petiolate, stem shorter than fork; fork III petiolate, stem longer than fork; Cu1 incomplete, not reaching wing margin; Cu1 and Cu2 intersecting near anastomosis; row of erect setae present along Cu2; A3 absent; crossveins forming a relatively linear transverse cord; discoidal cell longer than Rs vein. Hind wing margins nearly parallel, tapering only slightly past anastomosis; apical forks II and III present; Sc and R1 fused basally; A2 absent. Tibial spurs 1,4,4, foretibial spur extremely reduced and hairlike. Sixth sternal process short and digitate, apex rounded, associated with weak oblique apodeme posteriorly.

Male genitalia. Preanal and inferior appendages absent. Segment IX dorsally narrow, broad ventrally; anterior margin rounded; posterolateral margin membranous or very lightly sclerotized; sternum IX without modification. Tergum X incompletely fused to tergum IX with membrane or lightly sclerotized region ventrolaterally; dorsomesal margin with single, downturned, elongate process; dorsolateral margin irregular and setose; ventrolateral margin with paired subtriangular setose process directed posteriorly. Parameres present, paired, arising ventrobasally and fused to phallobase, sclerotized and rod-like, slender and elongate, sinuous, strongly downturned basally, distal portion slightly upturned and broader, directed posteriorly, apex pointed. Phallobase reduced, lightly sclerotized. Phallicata sinuous, with lightly sclerotized base, distal portion membranous, with pair of broad, sclerotized wing-like lateral flanges. Endophallus membranous, enlarged and convoluted when invaginated, with 3 upper lobes and 1 large lower lobe.

**Figure 12.**
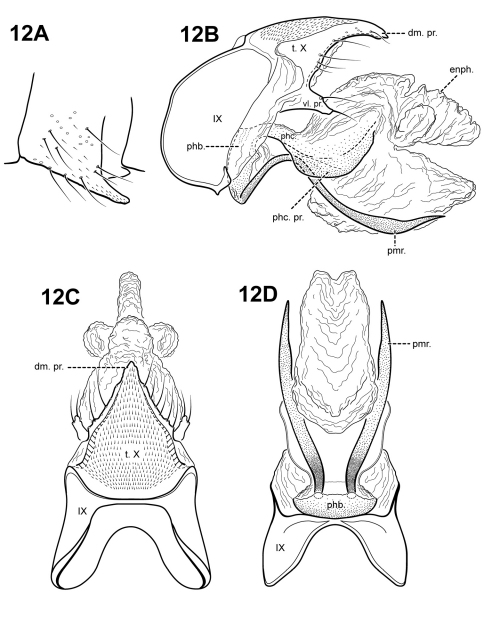
*Itauara guarani* (Angrisano, 1993) (UMSP000086361). Male genitalia: (**A**) Sternum VI process; ttt (**B**) lateral; (**C**)dorsal; (**D**) ventral. Abbreviations: dm. pr. = dorsomesal process; enph. = endophallus; phb = phallobase; phc. = phallicata; phc. pr. = phallicata process; pmr . = paramere; t. X = tergum X; vl. pr. = ventrolateral process.

#### Material examined.

**Holotype male: ARGENTINA: Misiones:** Departmento Belgrano, Rio Urugua-í (Releveamiento Faunístico Urugua-í [segundo campaña]) (UMSP000211316) (MACN).

**Allotype female: ARGENTINA: Misiones:** same data as holotype (MACN).

**Paratypes: ARGENTINA: Misiones:** same data as holotype – 3 males, 2 females (MACN).

The genitalia of the holotype and allotype were reported missing (E. Angrisano, *personal communication*).

### Itauara guyanensis

Robertson & Holzenthal sp. n.

urn:lsid:zoobank.org:act:3CD78294-B5E5-4191-A04E-23082102925F

http://species-id.net/wiki/Itauara_guyanensis

Fig. 2B 3B 13A–C 

#### Description. 

*Itauara guyanensis* has distinct extremely sinuous, almost corkscrew-shaped, parameres. The phallicata is short, sclerotized, and upturned apically and with 2 pointed lateral processes. The species can also be recognized by the thumb-like shape of the inferior appendage process and the presence of a bifid apical process in the endophallus. *Itaura guyanensis* is somewhat similar to *Itaura jamesii* sp. n., and resembles that species in the shape of the inferior appendage process and sinuous parameres. The 2 species can be easily separated by differences in the shape of tergum X and the phallicata. The species *Itaura alexanderi* sp. n., *Itaura emilia* sp. n., and *Itaura stella* sp. n., also have rather sinuous parameres, but differ in the shape of the phallicata, tergum X, and several other characters.

Adult. Body, wings, and appendages pale or tawny brown in alcohol. Forewing narrow past anastomosis, apex acute. Forewing venation incomplete, with apical forks I, II, and III present; Sc and R1 distinct along their entire lengths; fork I sessile; fork II petiolate, stem about the same length as fork; fork III petiolate, stem longer than fork; Cu1 complete, reaching wing margin; Cu1 and Cu2 intersecting near anastomosis; row of erect setae present along Cu2; A3 absent; crossveins forming a relatively linear transverse cord; discoidal cell longer than Rs vein. Hind wing narrow and slightly scalloped past anastomosis; apical fork II present; Sc and R1 fused basally; A2 absent. Tibial spurs 1,3,4, foretibial spur extremely reduced and hairlike. Sixth sternal process short and digitate, apex attenuate and pointed, associated with strong oblique apodeme posteriorly.

Male genitalia. Preanal appendages absent. Segment IX dorsally narrow, broad medially and ventrally; anterior margin rounded; posterolateral margin membranous or very lightly sclerotized; sternum IX without modification. Tergum X incompletely fused to tergum IX with membrane or lightly sclerotized region ventrolaterally; dorsomesal margin with single, downturned, elongate process; dorsolateral margin with paired small, down-turned, finger-like process; ventrolateral margin with an outer pair of subquadrate setose processes directed posteriorly, and an inner pair of subtriangular processes directed posteroventrally. Inferior appendages present as single thumb-like setose process, broadest at base and fused to phallobase ventrobasally. Parameres present, paired, inserted in membranous lobe, arising laterally from endotheca, sclerotized and rod-like, extremely sinuous, corkscrew-shaped, apex pointed. Phallobase reduced, lightly sclerotized with phallic shield. Phallicata forming a rather short sclerotized dorsal sheath extending from phallobase, straight basally and medially, bent sharply upward, medially with pair of lightly slerotized lateral flanges with pointed apices projecting posteriorly. Endophallus membranous, apically with sclerotized bifid process.

**Figure 13.**
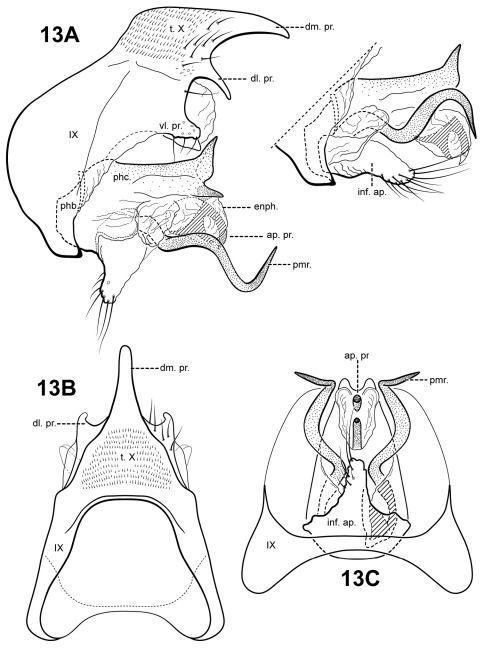
*Itauara guyanensis* sp. n. Male genitalia, everted (UMSP000210959) and non-everted (UMSP000210962). (**A**) lateral: left, everted; inset, non-everted (**B**) dorsal; (**C**) ventral. Abbreviations: ap. pr. = apical process; dl. pr. = dorsolateral process; dm. pr. = dorsomesal process; enph. = endophallus; inf. ap. = inferior appendage process; phb. = phallobase; phc. = phallicata; pmr . = paramere; t. X = tergum X; vl. pr. = ventrolateral process.

#### Material examined.

**Holotype male: GUYANA:** **Dubulay Ranch:** Warniabo Cr., 05°39'48"N, 057°53'24"W, 14–19.iv.1995 (O.S. Flint) (UMSP000210959) (NMNH).

**Paratypes: GUYANA: Dubulay Ranch:** Aramatani Cr., 05°39'24"N, 057°55'30"W, 15–18.iv.1995 (O.S. Flint) — 2 males, 2 females (NMNH).

#### Etymology. 

This species is named for the country of Guyana, where the specimens were collected.

### Itauara jamesii

Robertson & Holzenthal sp. n.

urn:lsid:zoobank.org:act:9EAE24A5-16C2-4B9F-8BDD-42976CE0E569

http://species-id.net/wiki/Itauara_jamesii

Fig. 14A–C 

#### Description. 

*Itauara jamesii* is known only from the male holotype, and its relationship to other species in not immediately evident. The parameres have a rather asymmetrical aspect, but it is possible that this particular specimen is distorted. The species has an inferior appendage process like several other species, but has a distinct subtriangular shape. The dorsomesal margin of tergum X is bifid, each half a small setose protuberance. *Itauara peruensis* sp. n., also has a bifid dorsomesal margin, but in that species, it appears as a prominent process with pointed apices.

Adult. Body, wings, and appendages pale or tawny brown, often intermingled with rufous or golden hairs, tibia and tarsi tawny brown. Wings with conspicuous white spot at the arculus and faint transverse line along anastomosis. Forewing slightly broader past anastomosis, but with margins nearly parallel, apex subacute. Forewing venation incomplete, with apical forks I, II, and III present; Sc and R1 distinct along their entire lengths; fork I sessile; fork II petiolate, stem shorter than fork; fork III petiolate, stem longer than fork; Cu1 complete, reaching wing margin; Cu1 and Cu2 intersecting near anastomosis; row of erect setae present along Cu2; A3 absent; crossveins forming a relatively linear transverse cord; discoidal cell longer than Rs vein. Hind wing margins nearly parallel, tapering only slightly past anastomosis; apical forks II and V present; Sc and R1 fused basally; A2 absent. Tibial spurs 1,4,4, foretibial spur extremely reduced and hairlike. Sixth sternal process thumb-like, apex rounded, associated with weak oblique apodeme posteriorly.

Male genitalia. Preanal and inferior appendages absent. Segment IX dorsally and ventrally narrow, broad medially; anterior margin rounded; posterolateral margin membranous or very lightly sclerotized; sternum IX without modification. Tergum X incompletely fused to tergum IX with membrane or lightly sclerotized region ventrolaterally; dorsomesal margin bifid and slightly produced, each half small, setose, with a rounded apex; dorsolateral margin without processes; ventrolateral margin with paired, broad, semi-circular setose flange-like process. Inferior appendages present as single, broad, subtriangular setose process, fused to phallobase ventrobasally, bearing small digitate lobes ventrolaterally, each bearing a seta. Parameres present, paired, inserted in membranous lobe, arising laterally from endotheca, sclerotized and rod-like, extremely sinuous, seemingly asymmetrical, apex pointed. Phallobase reduced, lightly sclerotized with phallic shield. Phallicata forming a long sclerotized dorsal sheath extending from phallobase, broadest basally, bent upward at middle, with paired sclerotized concave discs arising basodorsally and forming a connection with posterior margin of segment IX. Endophallus membranous, enlarged and convoluted when invaginated.

**Figure 14.**
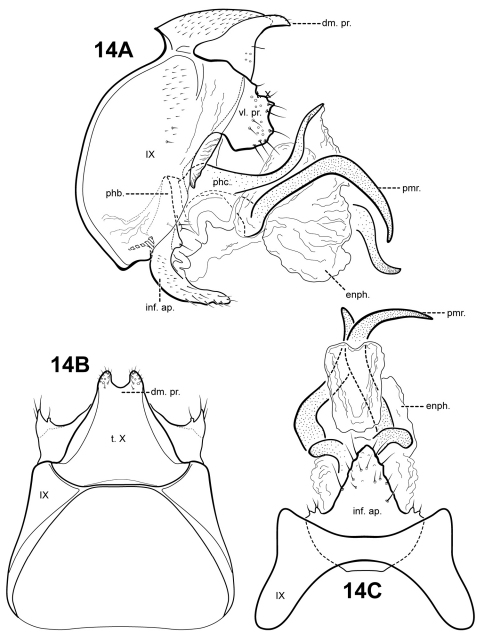
*Itauara jamesii* sp. n. (UMSP000087916). Male genitalia (**A**) lateral; (**B**) dorsal; (**C**) ventral. Abbreviations: dm. pr. = dorsomesal process; enph. = endophallus; enph. pr. = endophallic process; inf. ap. = inferior appendage process; phb. = phallobase; phc. = phallicata; pmr . = paramere; t. X = tergum X; vl. pr. = ventrolateral process.

#### Material examined.

**Holotype male: BRAZIL: Minas Gerais:** trib. to Rio do Salto, Ibitipoca, Fazenda Engenho, 21°44'06"S, 043°53'56"W, 875, 11–14.iii.2002 (Holzenthal, Blahnik, Paprocki, Prather) (UMSP000087916) (MZUSP).

#### Etymology.

We are delighted to name this species for the senior author’s father, James Gardner Robertson.

### Itauara julia

Robertson & Holzenthal sp. n.

urn:lsid:zoobank.org:act:5DE975A9-37FA-4BB0-9A52-662621648658

http://species-id.net/wiki/Itauara_julia

Fig. 1 3C 15A–C 

#### Description. 

This species is distinct in having a curved, spatulate ventral process in the endophallus and having a phallicata that is not continuous with the phallobase. The parameres of *Itaura julia* are strongly bent dorsally at the base, and terminate in a sharp, downturned point. The parameres curve upward, are fused to and continuous with the phallobase, and arise ventrobasally. The parameres of *Itaura blahniki* sp. n., *Itaura rodmani* sp. n., and *Itaura tusci* sp. n., are similarly structured. However, in these species, the parameres are not as abruptly bent.

Adult. Body, wings, and appendages pale or tawny brown, often intermingled with rufous or golden hairs, tibia and tarsi tawny brown. Wings with conspicuous white spot at the arculus. Forewing slightly broader past anastomosis, but with margins nearly parallel, apex rounded. With apical forks I, II, and III present; Sc and R1 distinct along their entire lengths; fork I sessile; fork II sessile; fork III petiolate, stem shorter than fork; Cu1 complete, reaching wing margin; Cu1 and Cu2 intersecting near anastomosis; row of erect setae present along Cu2; A3 absent; crossveins forming a relatively linear transverse cord; discoidal cell longer than Rs vein. Hind wing margins nearly parallel, tapering only slightly past anastomosis; apical forks II, III, and V present; Sc and R1 converging near wing margin; A2 absent. Tibial spurs 1,4,4, foretibial spur extremely reduced and hairlike. Sixth sternal process thumb-like, apex attenuate and pointed, associated with weak oblique apodeme posteriorly.

Male genitalia. Preanal and inferior appendages absent. Segment IX dorsally narrow, broad ventrally; anterior margin relatively straight from dorsum to medial area, ventral portion blunt; posterolateral margin membranous or very lightly sclerotized; sternum IX without modification. Tergum X incompletely fused to tergum IX with membrane or lightly sclerotized region ventrolaterally; dorsomesal margin straight, without processes; dorsolateral margin with several small irregular setose processes; ventrolateral margin with paired subquadrate setose process projecting ventrally. Parameres present, paired, arising ventrobasally and fused to phallobase, sclerotized and rod-like, slender and elongate, strongly bent upward basally, apex pointed and downturned, ventrobasally with a small patch of setae. Phallobase reduced, lightly sclerotized. Phallicata forming a long sclerotized dorsal sheath, mostly straight, broadest basally, apex with apicomesal point and 2 lateral downturned points. Endophallus membranous, enlarged and convoluted when invaginated, ventrally with a curved spatulate process.

**Figure 15.**
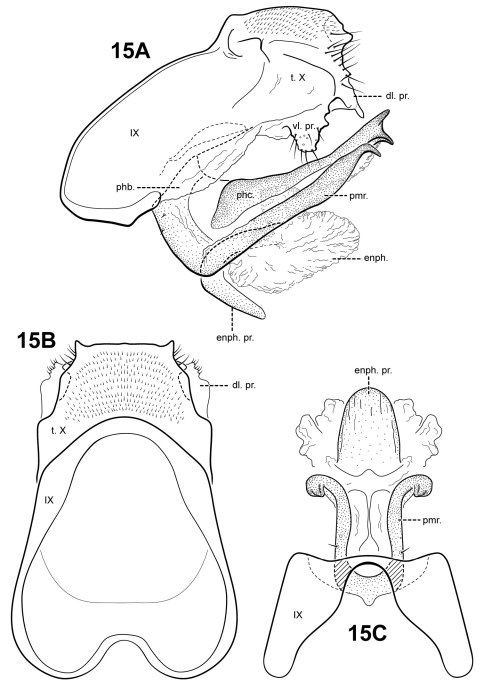
*Itauara julia* sp. n. Male genitalia: Male genitalia (**A**) lateral; (**B**) dorsal; (**C**) ventral. Abbreviations: dl. pr. = dorsolateral process; enph. = endophallus; enph. pr. = endophallic process; phb. = phallobase; phc. = phallicata; pmr . = paramere; t. X = tergum X; vl. pr. = ventrolateral process.

#### Material examined.

 **Holotype male: BRAZIL: Rio de Janeiro:** Parque Nacional do Itatiaia, Rio Campo Belo, trail to Veu da Noiva, 22°25'42"S, 044°37'10"W, 1310 m, 5.iii.2002 (Holzenthal, Blahnik, Paprocki, Prather) (UMSP000069560) (MZUSP).

**Paratypes: BRAZIL: Rio de Janeiro:** Parque Nacional do Itatiaia, same data as holotype — 9 males, 5 females (UMSP); same, 24.ix.2001 (Holzenthal, Blahnik, Neto, Paprocki) — 17 males, 8 females (UMSP); Rio Taquaral, 22°27'15"S, 044°36'34"W, 1300 m, 22–23.ix.2001 (Holzenthal & Blahnik) — 20 males; Rio Campo Belo, 22°27'02"S, 044°36'49"W, 1300 m, 23.ix.2001 (Holzenthal, Blahnik, Neto, Paprocki) — 28 males, 17 females (NMNH, UMSP); 7.iii.2002 (Holzenthal, Blahnik, Paprocki, Prather) —24 males, 46 females (MZUSP, UMSP).

#### Etymology.

We are delighted to name this species for Julie Martinez, who rendered the beautiful color plate of this species.

### Itauara lucinda

Robertson & Holzenthal sp. n.

urn:lsid:zoobank.org:act:F8FA9F0B-D595-4FDC-92D8-7F64F183E91D

http://species-id.net/wiki/Itauara_lucinda

Fig. 16A–C 

#### Description. 

This species is similar to *Itauara alexanderi* sp. n., *Itaura emilia* sp. n., and *Itaura stella* sp. n., as discussed under each of those species. Each of these species possess an inferior appendage process, a dorsomesal process on tergum X, and rather sinuous parameres. Among these species, *Itaura lucinda* is distinct in having forked parameres and a non-bifid inferior appendage process.

Adult. Body, wings, and appendages fuscous, intermingled with rufous or golden hairs, tibia and tarsi tawny brown. Wings with white transverse line along anastomosis. Forewing slightly broader past anastomosis, but with margins nearly parallel, apex subacute. Forewing venation incomplete, with apical forks I, II, and III present; Sc and R1 distinct along their entire lengths; fork I sessile; fork II petiolate, stem about the same length as fork; fork III petiolate, stem longer than fork; Cu1 complete, reaching wing margin; Cu1 and Cu2 intersecting near anastomosis; row of erect setae present along Cu2; A3 absent; discoidal cell longer than Rs vein. Hind wing margins nearly parallel, tapering only slightly past anastomosis; apical forks II and V present; Sc and R1 fused basally; A2 absent. Tibial spurs 1,4,4, foretibial spur extremely reduced and hairlike. Sixth sternal process thumb-like, apex attenuate and pointed, associated with oblique apodeme posteriorly.

Male genitalia. Preanal appendages absent. Segment IX dorsally narrow, broad medially and ventrally; anterior margin rounded; posterolateral margin membranous or very lightly sclerotized; sternum IX without modification. Tergum X incompletely fused to tergum IX with membrane or lightly sclerotized region ventrolaterally; dorsomesal margin with single, downturned, elongate process; dorsolateral margin without processes; ventrolateral margin with paired, broad flange-like setose process with small upper lobe and larger subquadrate lower lobe. Inferior appendages present as single, broad, subquadrate setose process, broadest basally, fused to phallobase ventrobasally, with 2 pairs of small digitate lobes ventrolaterally, each bearing a seta. Parameres present, paired, inserted in membranous lobe, arising laterally from endotheca, sclerotized and rod-like, bifid, with short lower process and longer, slightly medially bent upper process, directed posteriorly, apices pointed. Phallobase reduced, lightly sclerotized. Phallicata forming a long, straight sclerotized dorsal sheath extending from phallobase. Endophallus membranous, enlarged and convoluted when invaginated, with 1 upper and 1 lower lobe.

**Figure 16.**
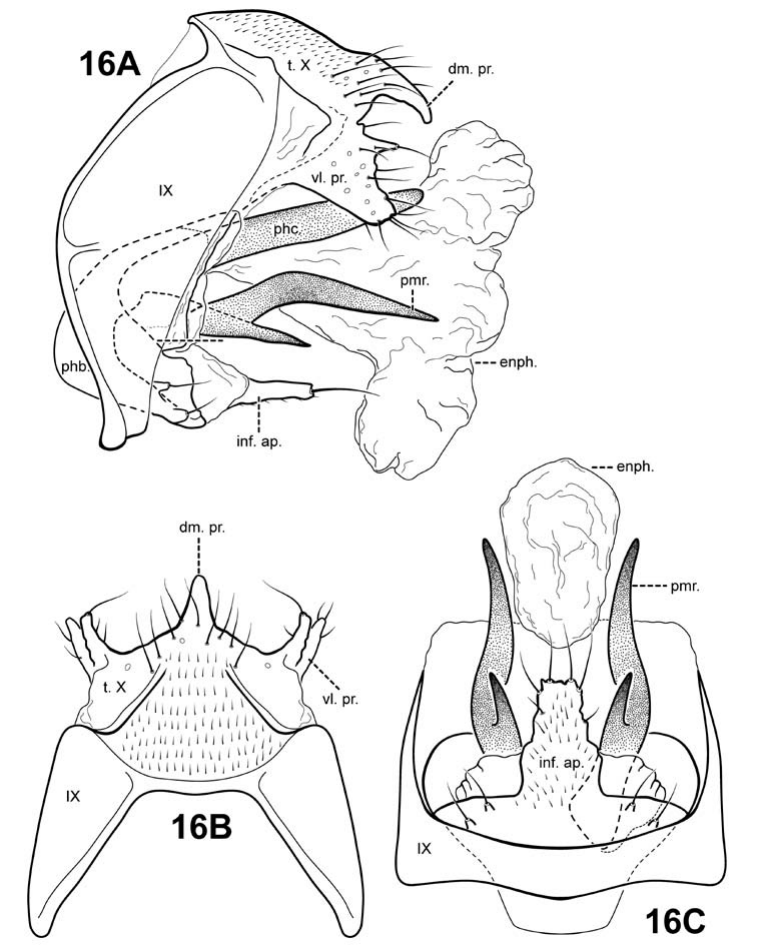
*Itauara lucinda* sp. n. (UMSP000052593). Male genitalia (**A**) lateral; (**B**) dorsal; (**C**) ventral. Abbreviations: dm. pr. = dorsomesal process; enph. = endophallus; inf. ap. = inferior appendage process; phb. = phallobase; phc. = phallicata; pmr . = paramere; t. X = tergum X; vl. pr. = ventrolateral process.

#### Material examined.

**Holotype male: BRAZIL: Minas Gerais:** Parque Nacional do Caparaó, small trib. to Rio Caparaó, Vale Verde, 20°25'02"S, 041°50'46"W, 1350, 12–14.iii.2002 (R.W. Holzenthal) (UMSP000052593) (MZUSP).

**Paratype:** **BRAZIL: Minas Gerais:** same data as holotype — 1 female (UMSP).

#### Etymology.

We are delighted to name this species for the senior author’s daughter, Lucinda Grace Thompson.

### Itauara ovis

Robertson & Holzenthal sp. n.

urn:lsid:zoobank.org:act:B534B28B-C523-4B04-9F33-FDE480C6D2BE

http://species-id.net/wiki/Itauara_ovis

Fig. 17A–C 

#### Description. 

Perhaps the most notable feature of this species is the extremely curved, ram-like shaped parameres. *Itauara spiralis* sp. n., also has highly curved, spiral shaped parameres, but in *Itaura spiralis*, the paramere is curved along the entire length of the paramere, whereas in *Itaura ovis*, the paramere is curved basally, but straight distally. The 2 species differ in other respects, including the shape of the inferior appendage process. In *Itaura ovis*, the inferior appendage process is elongate, and rather inflated apically; in *Itaura spiralis*, the inferior appendage process is bifid. The 2 species also differ in the shape of tergum X. In *Itaura ovis*, tergum X is slightly notched apicomesally and has 2 pairs of rather small, subtriangular ventrolateral processes. In *Itaura spiralis*, tergum X is not notched, but has a pair of very long, finger-like dorsomesal processes and a pair of very broad, irregular ventrolateral processes. The phallicata of *Itaura ovis* is also quite distinct, being rather broad, and saddle-shaped, with a dorsobasal hump and upturned apex.

Adult. Body, wings, and appendages fuscous, intermingled with rufous or golden hairs, tibia and tarsi tawny brown. Wings often with a few pale cream-colored or white hairs at arculus. Forewing relatively narrow, with margins nearly parallel, apex subacute. Forewing venation incomplete, with apical forks I, II, and III present; fork I sessile; fork II petiolate, stem about the same length as fork; fork III petiolate, stem longer than fork; Cu1 complete, reaching wing margin; Cu1 and Cu2 intersecting near anastomosis; row of erect setae present along Cu2; A3 absent; crossveins forming a relatively linear transverse cord; discoidal cell longer than Rs vein. Hind wing narrow and slightly scalloped past anastomosis; apical forks II and V present; Sc and R1 fused basally; A2 absent. Tibial spurs 1,4,4, foretibial spur extremely reduced and hairlike. Sixth sternal process short and digitate, apex attenuate and pointed, associated with strong oblique apodeme posteriorly.

Male genitalia. Preanal appendages absent. Segment IX ventrally narrow, broad medially; anterior margin rounded; posterolateral margin lightly sclerotized; sternum IX without modification. Tergum X incompletely fused to tergum IX with membrane or lightly sclerotized region ventrolaterally; dorsomesal margin subtriangular, slightly produced with small cleft; dorsolateral margin without processes; ventrolateral margin with an outer pair of subtriangular setose processes directed ventrally, and an inner pair of subtriangular processes directed posteroventrally. Inferior appendages present as single, rather elongate setose process produced mesally, apex broad and slightly irregular, fused to phallobase ventrobasally. Parameres present, paired, inserted in membranous lobe, arising laterally from endotheca, sclerotized and rod-like, ram-like, curving 360 degrees at base, distal portion straight, directed posteriorly, apex pointed. Phallobase extremely reduced and difficult to discern. Phallicata forming a saddle-shaped sclerotized dorsal sheath, with dorsal hump basally, distal portion curving upward. Endophallus membranous, enlarged and convoluted when evaginated, with pair of elongate lateral sclerites ventrally.

**Figure 17.**
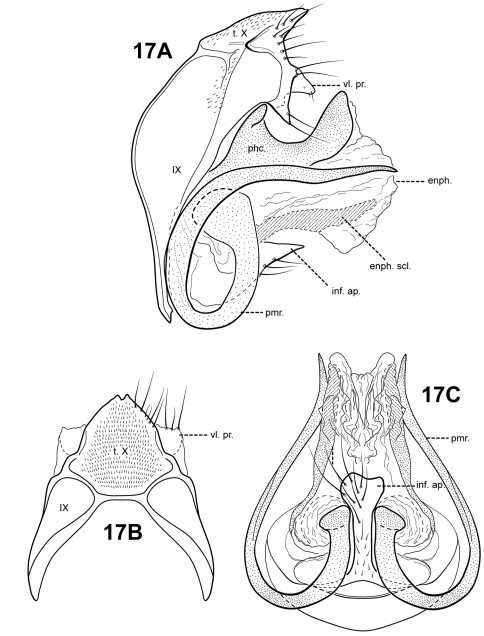
*Itauara ovis* sp. n. (UMSP000118534) Male genitalia. (**A**) lateral; (**B**) dorsal; (**C**) ventral. Abbreviations: enph. = endophallus; enph. scl. = endophallic sclerite; inf. ap. = inferior appendage process; phc. = phallicata; pmr . = paramere; t. X = tergum X; vl. pr. = ventrolateral process.

#### Material examined.

**Holotype male: GUYANA: Kanuku Mountains:** Kumu River & Falls, 03°15'54"N, 059°43'30"W, 28–30.iv.1995 (O.S. Flint) (UMSP000118534) (NMNH)

**Paratypes: VENEZUELA: Bolivar:** La Escalera, 108 km. S Rio Cuyuni, 11–12.ii.1976 (C. & O. Flint) — 5 males (NMNH).

#### Etymology.

The name *ovis*, comes from the Latin for sheep, and is suggested by the shape of the parameres, which are reminiscent of a ram’s horn.

### Itauara peruensis

Robertson & Holzenthal sp. n.

urn:lsid:zoobank.org:act:0F3E4131-B78D-470A-9DA5-83228D16BEC6

http://species-id.net/wiki/Itauara_peruensis

Fig. 18A–C 

#### Description. 

This species is distinct in having a lightly sclerotized endophallus. Ventrally, the endophallus is membranous, but in lateral view, it has the appearance of being entirely sclerotized. Another unique feature is the prominent bifid dorsomesal process of tergum X. *Itauara peruensis* has a rather elongate inferior appendage process like *Itaura ovis*, but it is not inflated apically like that species. The species also differ in the shape of the parameres; those of *Itaura peruensis* are nearly straight, while those of *Itaura ovis* are spiral-shaped.

Adult. Body, wings, and appendages pale or tawny brown in alcohol. Forewing narrow past anastomosis, apex acute. Forewing venation incomplete, with apical forks I, II, and III present; Sc and R1 distinct along their entire lengths; fork I sessile; fork II petiolate, stem about the same length as fork; fork III petiolate, stem longer than fork; Cu1 complete, reaching wing margin; Cu1 and Cu2 intersecting near anastomosis; row of erect setae present along Cu2; A3 absent; crossveins forming a relatively linear transverse cord; discoidal cell longer than Rs vein. Hind wing narrow and slightly scalloped past anastomosis; apical fork II present; Sc and R1 fused basally; A2 absent. Tibial spurs 1,4,4, foretibial spur extremely reduced and hairlike. Sixth sternal process short and digitate, apex attenuate and pointed, associated with strong oblique apodeme posteriorly.

Male genitalia. Preanal appendages absent. Segment IX ventrally narrow, broad medially; anterior margin rounded; posterolateral margin membranous or very lightly sclerotized; sternum IX without modification. Tergum X incompletely fused to tergum IX with membrane or lightly sclerotized region ventrolaterally; dorsomesal margin with bifid process, each half with a pointed apex; dorsolateral margin slightly irregular, without processes; ventrolateral margin with paired subtriangular setose process directed ventrally. Inferior appendages present as single, narrow, rather short setose process produced mesally, fused to phallobase ventrobasally. Parameres present, paired, arising laterally from anterior portion of phallobase, sclerotized and rod-like, slender and elongate, straight, very slightly downturned, apex pointed. Phallobase reduced, lightly sclerotized. Phallicata forming a long sclerotized dorsal sheath, mostly straight, broadest basally, distal portion very slightly upturned. Endophallus lightly sclerotized tubular structure, ventrally with membranous folds, apically with small phallotremal sclerite.

**Figure 18.**
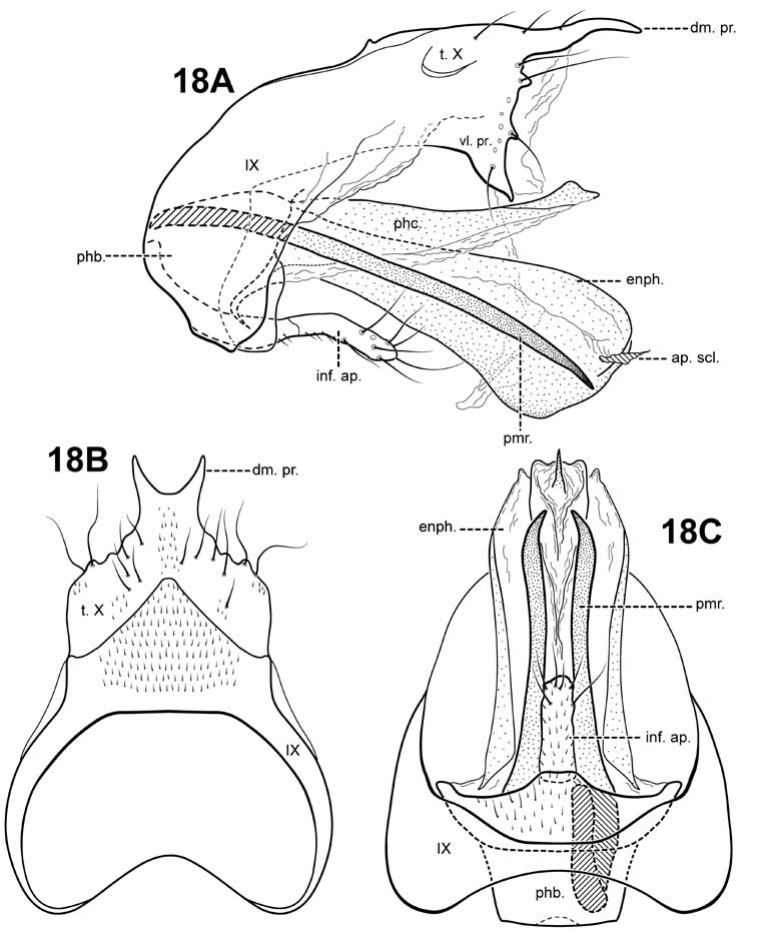
*Itauara peruensis* sp. n. (UMSP000210957). Male genitalia (**A**) lateral; (**B**) dorsal; (**C**) ventral. Abbreviations: ap. scl. = apical sclerite; dm. pr. = dorsomesal process; enph. = endophallus; inf. ap. = inferior appendage process; phb. = phallobase; phc. = phallicata; pmr . = paramere; t. X = tergum X; vl. pr. = ventrolateral process.

#### Material examined.

**Holotype male: PERU: Madre de Dios:** Manu Biosphere Reserve, Pakitza Biological Station, Trail 2, 1st stream, 12°07'00"S, 070°58'00"W, 250 m, 14–23.ix.1988 (Flint & Adams) (UMSP000210957) (NMNH)

**Paratypes:** **PERU: Madre de Dios**: same data as holotype — 7 males, 21 females; same, 17–20.ix.1988 (Flint & Adams) — 1 female (NMNH).

#### Etymology.

This species is named for the country of Peru, where the specimens were collected.

### Itauara plaumanni

(Flint 1974)

http://species-id.net/wiki/Itauara_plaumanni

Fig. 19A–D

plaumanni  (Flint), 1974: 7 [Type locality: Brazil, Santa Catarina, Nova Teutonia; NMNH; in *Antoptila*]. – Angrisano, 1993: 59 [distribution] – Flint, Holzenthal, and Harris, 1999:74 [to *Itauara*].

#### Description. 

*Itauara plaumanni* can be recognized by the irregular lobe-like shape of the lateral flanges on the phallicata. Additionally, this species has a rather elongate segment IX and tergum X. The profile of tergum X resembles that of *Itaura brasiliana*, but *Itaura plaumanni* has fewer lateral processes than that species. *Itauara plaumanni* has parameres similar in shape to those of *Itaura guarani*, yet these species are easily separated by differences in the shape of the lateral flanges of the phallicata, and tergum X.

Adult. Body, wings, and appendages pale or tawny brown in alcohol. Forewing slightly broader past anastomosis, but with margins nearly parallel, apex rounded. Forewing venation incomplete, with apical forks I, II, and III present; Sc and R1 distinct along their entire lengths; fork I sessile; fork II petiolate, stem shorter than fork; fork III petiolate, stem about the same length as fork; Cu1 complete, reaching wing margin; Cu1 and Cu2 intersecting near anastomosis; row of erect setae present along Cu2; A3 absent; crossveins forming a relatively linear transverse cord; discoidal cell longer than Rs vein. Hind wing margins nearly parallel, tapering only slightly past anastomosis; apical forks II, III, and V present; Sc and R1 fused basally; A2 absent. Tibial spurs 1,4,4, foretibial spur extremely reduced and hairlike. Sixth sternal process short and digitate, apex attenuate and pointed.

Male genitalia. Preanal and inferior appendages absent. Segment IX relatively broad; anterior margin rounded; posterolateral margin membranous or very lightly sclerotized; sternum IX without modification. Tergum X incompletely fused to tergum IX with membrane or lightly sclerotized region ventrolaterally; dorsomesal margin slightly produced as small irregular point; dorsolateral margin with 2 pairs of processes, the upper an elongate subtriangular process slightly downturned, the lower a small lobe-like setose process; ventrolateral margin without processes. Parameres present, paired, arising ventrobasally and fused to phallobase, sclerotized and rod-like, slender and elongate, sinuous, downturned basally, distal portion slightly upturned, directed posteriorly and inward, apex pointed, ventrobasally with small patch of setae. Phallobase reduced, lightly sclerotized. Phallicata forming a long sclerotized dorsal sheath, slightly bent upward medially, with pair of irregular lobe-like lateral flanges projecting posteroventrally. Endophallus membranous, enlarged and convoluted when invaginated, with lightly sclerotized lobe ventrally and laterally, containing 2 small sclerites.

**Figure 19.**
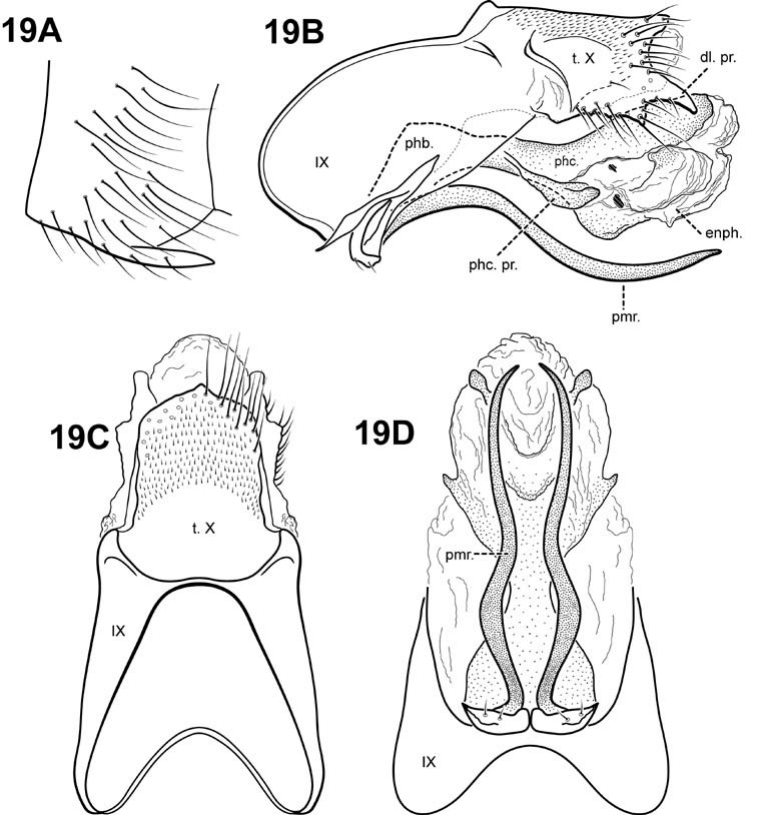
*Itauara plaumanni* (Flint, 1974) (UMSP000086359). Male genitalia: (**A**) Sternum VI process; ttt (**B**) lateral; (**C**) dorsal; (**D**) ventral. Abbreviations: dl. pr. = dorsolateral process; enph. = endophallus; phb = phallobase; phc. = phallicata; phc. pr. = phallicata process; pmr . = paramere; t. X = tergum X.

#### Material examined.

**Holotype male: BRAZIL: Santa Catarina:** Nova Teutonia, 27°03'00"S, 052°24'00"W, 1.ii.1964 (F. Plaumann) (UMSP000027160) (NMNH).

**Paratypes: BRAZIL: Santa Catarina:** same data as holotype, 1.ix.1963 (F. Plaumann) — 1 male (NMNH), same, 1.xi.1963 (F. Plaumann) — 7 males (NMNH); same, Nova Teutonia, 27°11'00"S, 052°23'00"W, 300–500 m, 1.i.1963 (F. Plaumann) — 1 male, (NMNH), same, 1.i.1964 (F. Plaumann) — 7 males (NMNH).

Additional material examined: **ARGENTINA: Misiones:** Cataratas del Iguazú, 14.x.1985 — 2 males (MACN); **Salto:** Salto Grande, cascada, 10.xi.1955 (C.S. Carbonell) — 46 males (MACN); **URUGUAY: Artigas:** San Gregorio, 30°33'00"S, 057°52'00"W (Carbonell, Mesa, & San Martin) — 1 male (MACN); Orillas Rio Uruguay (Carbonell, AM, PSM) — 1 male, 3 females (MACN); **Paysandu:** Sta. Rita, Orilla Rio Uruguay, 32°07'00"S, 058°09'00"W, 8.xii.1955 (C.S. Carbonell) — 10 males (MACN); 1.xii.1959 (C.S. Carbonell) — 1 male (MACN).

### Itauara rodmani

Robertson & Holzenthal sp. n.

urn:lsid:zoobank.org:act:B5D07A37-7980-4300-B3E1-C856A34EA331

http://species-id.net/wiki/Itauara_rodmani

Fig. 20A–D 

#### Description. 

This species is very similar to *Itaura tusci* sp. n., which also has very long, upturned, tusk-like parameres and a strongly upturned phallicata. The 2 species can be separated based on the shape of the phallicata process, which is pointed and blade-like in *Itaura tusci* and rounded or blunt in *Itaura rodmani*. The phallicata is also more sclerotized in *Itaura rodmani*. Additionally, the dorsomesal margin of tergum X is irregular, with several small setose processes in *Itaura tusci*, whereas in *Itaura rodmani*, the dorsomesal margin is rather smooth and triangular. The ventrolateral processes of the 2 species also differ: *Itaura tusci* has a small upper and more elongate lower process; *Itaura rodmani* has a single, short, digitate process. *Itaura blahniki* also has upturned, tusk-like parameres, but is easily distinguished from *Itaura rodmani* based on differences in tergum X and the phallicata. *Itauara blahniki* and *Itaura blahniki* sp. n., also has tusk-like parameres, but they are not as long and curved as *Itaura rodmani*.

Adult. Body, wings, and appendages pale or tawny brown in alcohol. Wings with conspicuous white spot at the arculus. Forewing slightly broader past anastomosis, but with margins nearly parallel, apex subacute. Forewing venation incomplete, with apical forks I, II, and III present; Sc and R1 distinct along their entire lengths; fork I sessile; fork II petiolate, stem shorter than fork; fork III petiolate, stem about the same length as fork; Cu1 incomplete, not reaching wing margin; Cu1 and Cu2 intersecting near anastomosis; row of erect setae present along Cu2; A3 absent; crossveins forming a relatively linear transverse cord; discoidal cell longer than Rs vein. Hind wing margins nearly parallel, tapering only slightly past anastomosis; apical forks II, III, and V present; Sc and R1 fused basally; A2 absent. Tibial spurs 1,4,4, foretibial spur extremely reduced and hairlike. Sixth sternal process thumb-like, apex rounded, associated with weak oblique apodeme posteriorly.

Male genitalia. Preanal and inferior appendages absent. Segment IX relatively broad; anterior margin rounded; posterolateral margin membranous or very lightly sclerotized; sternum IX without modification. Tergum X incompletely fused to tergum IX with membrane or lightly sclerotized region ventrolaterally; dorsomesal margin subtriangular, very slightly downturned; dorsolateral margin with paired small, slightly down-turned, setose process; ventrolateral margin with an outer and inner pair of small setose processes directed posteriorly. Parameres present, paired, arising ventrobasally from fused endotheca and phallobase, sclerotized and rod-like, tusk-like, strongly curving upward, apex pointed. Phallobase reduced, lightly sclerotized with phallic shield. Phallicata forming a long sclerotized dorsal sheath extending from phallobase, strongly curving upward with apex directed dorsally, with pair of broad, sclerotized wing-like lateral flanges with rounded or subquadrate ventral margins. Endophallus membranous, enlarged and convoluted when invaginated, with 1 tubular upper lobe and 3 smaller lower lobes.

**Figure 20.**
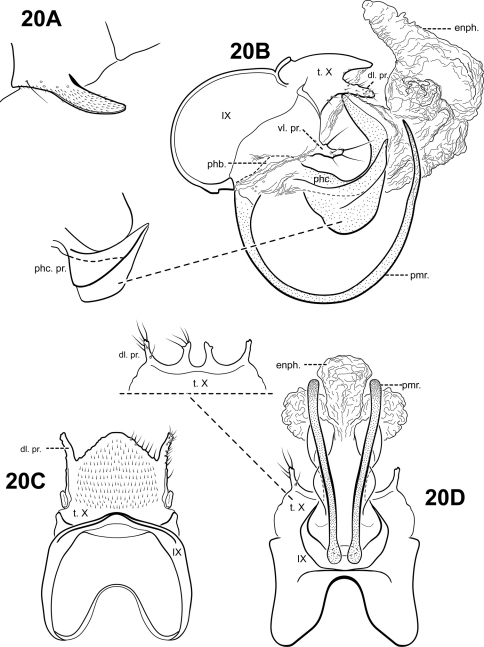
*Itauara rodmani* sp. n. (UMSP000081856). Male genitalia. (**A**) sternum VI process; (**B**) lateral; inset, variant (**C**) dorsal; (**D**) ventral; inset, tergum X. Abbreviations: dl. pr. = dorsolateral process; enph. = endophallus; phb = phallobase; phc. = phallicata; phc. pr. = phallicata process; pmr . = paramere; t. X = tergum X; vl. pr. = ventrolateral process.

#### Material examined.

**Holotype male: BRAZIL: Minas Gerais:** Corrego das Aguas Pretas & tribs., ca. 15 km S Aiuruoca, 22°03'42"S, 044°38'14"W, 1386 m, 21.xi.2001 (Holzenthal, Blahnik, Neto, Paprocki) (UMSP000081857) (MZUSP).

**Paratypes: BRAZIL: Minas Gerais:** same data as holotype — 6 females, 3 males (UMSP).

#### Etymology.

We are delighted to name this species for Dr. James Rodman, the NSF program director who initiated the Partnership for Enhancing Expertise in Taxonomy (PEET) program. The PEET program provides funding for the training of taxonomists of little known organisms. The senior author is grateful for the wonderful experience she had while participating in the PEET program as a doctoral student and the opportunity to study Trichoptera taxonomy.

### Itauara simplex

Robertson & Holzenthal sp. n.

urn:lsid:zoobank.org:act:E7688425-1DE8-4A29-8902-DCDCFC75B036

http://species-id.net/wiki/Itauara_simplex

Fig. 21A–C 

#### Description. 

This species can be recognized by its rather simple genitalic capsule. Tergum X is produced dorsomesally into a broad, elongate plate and has just one small ventrolateral process. The parameres are relatively short, straight basally, but slightly bent downward distally. The phallicata is short and very lightly sclerotized, and the endophallus is large and membranous, with 2 lateral patches or elongate setae apically. *Itauara guarani* also has a very lightly sclerotized phallicata, but the 2 species differ in the shape of the parameres and *Itaura guarani* has lateral flange-like processes on the phallicata.

Adult. Body, wings, and appendages fuscous, intermingled with rufous or golden hairs. Wings with conspicuous white spot at the arculus. Forewing slightly broader past anastomosis, but with margins nearly parallel, apex subacute. Forewing venation incomplete, with apical forks I, II, and III present; Sc and R1 distinct along their entire lengths; fork I petiolate, but with extremely short stem; fork II petiolate, stem about the same length as fork; fork III petiolate; stem longer than fork; Cu1 complete, reaching wing margin; Cu1 and Cu2 intersecting near anastomosis; row of erect setae present along Cu2; A3 absent; crossveins forming a relatively linear transverse cord; discoidal cell longer than Rs vein. Hind wing margins nearly parallel, tapering only slightly past anastomosis; apical fork II present; Sc and R1 fused basally; A2 absent. Tibial spurs 1,4,4, foretibial spur extremely reduced and hairlike. Sixth sternal process thumb-like, apex rounded, associated with weak oblique apodeme posteriorly.

Male genitalia. Preanal and inferior appendages absent. Segment IX dorsally narrow, broad medially and ventrally; anterior margin rounded; posterolateral margin lightly sclerotized; sternum IX without modification. Tergum X incompletely fused to tergum IX with membrane or lightly sclerotized region ventrolaterally; dorsomesal margin produced into a single broad, plate-like process; dorsolateral margin slightly irregular, without processes; ventrolateral margin with small, irregular, paired setose process. Parameres present, paired, arising ventrobasally from fused endotheca and phallobase, sclerotized and rod-like, slender and elongate, straight medially and basally, curving downward distally, directed posteroventrally, apex pointed. Phallobase reduced, lightly sclerotized with phallic shield. Phallicata short, with lightly sclerotized base, rugous medially, becoming membranous distally. Endophallus membranous, enlarged and convoluted when invaginated, with1 upper lobe and 2 lower lobes, with paired patch of elongate setae laterally.

**Figure 21.**
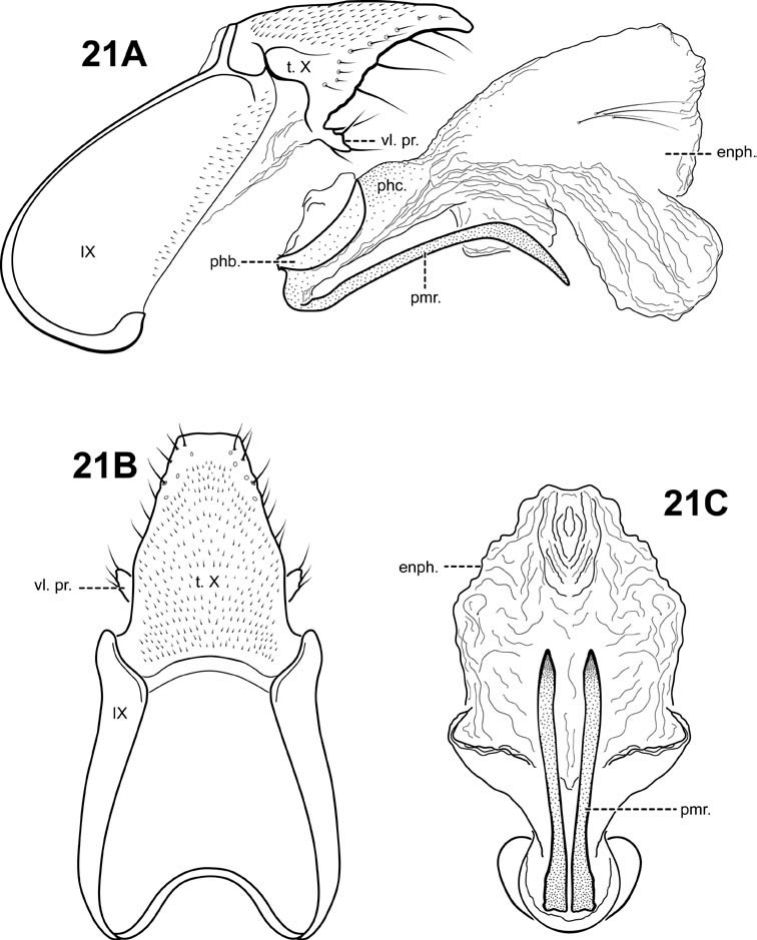
*Itauara simplex* sp. n. (UMSP000069700). Male genitalia: (**A**) lateral; (**B**) dorsal; (**C**) ventral. Abbreviations: enph. = endophallus; phb = phallobase; phc. = phallicata; pmr . = paramere; t. X = tergum X; vl. pr. = ventrolateral process.

#### Material examined.

 **Holotype male: BRAZIL: São Paulo:** Parque Nacional da Serra da Bocaina, Cachoeira dos Posses, 22°46'26"S, 044°36'15"W, 1250, 3.iii.2002 (Holzenthal, Blahnik, Paprocki, Prather) (UMSP000069700) (MZUSP).

**Paratype:** **BRAZIL: São Paulo:** same data as holotype — 1 female (UMSP).

#### Etymology.

This species is so named for the rather simple structure of the phallic apparatus and genital capsule.

### Itauara spiralis

Robertson & Holzenthal sp. n.

urn:lsid:zoobank.org:act:0568AD89-DD46-4FCB-87F4-0F9D884AEC04

http://species-id.net/wiki/Itauara_spiralis

Fig. 22A–C 

#### Description. 

This species is distinct in having a sclerotized, tubular phallicata, and an elongate, laterally compressed, dorsomesal spine. The phallicata in other species are less tubular, appearing as a dorsal sheath. This dorsal sheath was identified as a synapomorphy for the genus in a previous phylogenetic study of Protoptilinae (see Chapter 1, this work). *Itauara spiralis* was not included in that study, however, *Itaura spiralis* is placed in *Itauara* since it shares many other characteristics common to the genus such as an inferior appendage process, and a tergum X that is nearly identical to *Itaura bidentata* sp. n. and *Itaura unidentata* sp. n.

*Itauara spiralis* can be recognized by the extremely curved, spiral-shaped parameres. *Itaura ovis* sp. n., also has highly curved, spiral shaped parameres, but in *Itaura spiralis*, the paramere is curved along the entire length of the paramere, whereas in *Itaura ovis*, the paramere is curved basally, but straight distally. The 2 species differ in other respects, including the shape of the inferior appendage process, which is bifid in *Itaura spiralis* and inflated apically in *Itaura ovis*. The 2 species also differ in the shape of tergum X and the phallicata. Tergum X is very similar to those of *Itaura bidentata* and *Itaura unidentata*; all have elongate, finger-like dorsolateral processes and broad, irregular, setose ventrolateral processes. *Itauara spiralis* is distinguished from these other 2 species by having a bifid inferior appendage process, spiral-shaped parameres, and laterally compressed dorsomesal spine.

Adult. The only specimen of this species is in very poor condition. Therefore, head, thoracic, and wing characters could not be observed. However, the genitalia are intact.

Male genitalia. Preanal appendages absent. Segment IX ventrally narrow, broad medially; anterior margin rounded; posterolateral margin membranous or very lightly sclerotized; sternum IX without modification. Tergum X incompletely fused to tergum IX with membrane or lightly sclerotized region ventrolaterally; dorsomesal margin straight, shallowly excavate; dorsolateral margin with paired elongate, down-turned, finger-like process; ventrolateral margin with paired, very broad flange-like setose process consisting of several small irregular lobes. Inferior appendages present as apically bifid, setose process produced mesally, broadest at base and fused to phallobase ventrobasally. Parameres present, paired, arising laterally from endotheca, spiral-shaped, curving 360 degrees at base with curve continuing to apex, directed posteroventrally, apex pointed. Phallobase reduced, lightly sclerotized. Phallicata forming a short slerotized dorsal tube extending from phallobase, with a long, broad dorsomesal spine arising posteriorly to phallobase. Endophallus membranous, rather small, apically sharply bent downward, pointing anteroventrally.

**Figure 22.**
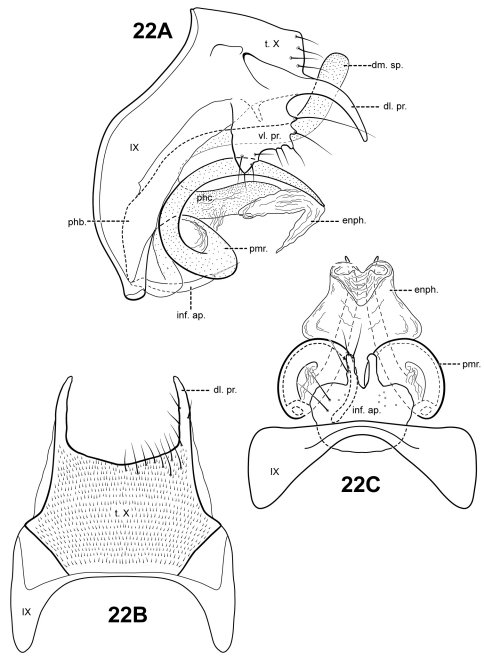
*Itauara spiralis* sp. n. (UMSP000210960). Male genitalia (**A**) lateral; (**B**) dorsal; (**C**) ventral. Abbreviations: dl. pr. = dorsolateral process; dm. sp. = dorsomesal spine; enph. = endophallus; inf. ap. = inferior appendage process; phb. = phallobase; phc. = phallicata; pmr . = paramere; t. X = tergum X; vl. pr. = ventrolateral process.

#### Material examined.

**Holotype male: GUYANA: Paramakatoi:** 04°42'00"N, 059°42'48"W, 24–25.viii.1997 (W.N. Mathis) (UMSP0000210960) (NMNH).

#### Etymology.

The name *spiralis* refers to the spiral form of the parameres.

### Itauara stella

Robertson & Holzenthal sp. n.

urn:lsid:zoobank.org:act:D8B2481D-60A2-42D3-A6A7-3D904B397203

http://species-id.net/wiki/Itauara_stella

Fig. 23A–C 

#### Description. 

This species is associated with *Itauara alexanderi* sp. n., *Itaura emilia* sp. n., and *Itaura lucinda* sp. n., as discussed under each of those species. Each of these species possess an inferior appendage process, a dorsomesal process on tergum X, and rather sinuous parameres. Of these species, *Itaura stella* is most similar to *Itaura alexanderi*. Both of these species have similarly shaped elongate dorsomesal processes and broad ventrolateral processes of tergum X. Both also have apically bifid inferior appendage processes. *Itauara stella* can be distinguished from by the length of the parameres; those of *Itaura stella* are longer and more strongly directed laterally than those of *Itaura alexanderi*. Additionally, the inferior appendage process of *Itaura alexanderi* is broader than that of *Itaura stella*. *Itauara stella* can be differentiated from *Itaura emilia* by the shape of the dorsomesal process and from *Itaura lucinda* by the shape of the parameres and inferior appendage process.

Adult. Body, wings, and appendages pale or tawny brown in alcohol. Wings with white transverse line along anastomosis. Forewing slightly broader past anastomosis, but with margins nearly parallel, apex rounded. Forewing venation incomplete, with apical forks I, II, and III present; Sc and R1 distinct along their entire lengths; fork I sessile; stem about the same length as fork; fork III petiolate, stem about the same length as fork; Cu1 complete, reaching wing margin; Cu1 and Cu2 intersecting near anastomosis; row of erect setae present along Cu2; A3 absent; crossveins forming a relatively linear transverse cord. Hind wing margins nearly parallel, tapering only slightly past anastomosis; apical forks II and V present; Sc and R1 fused basally; A2 absent. Tibial spurs 1,4,4, foretibial spur extremely reduced and hairlike. Sixth sternal process thumb-like, apex attenuate and pointed, associated with weak oblique apodeme posteriorly.

Male genitalia. Preanal appendages absent. Segment IX ventrally narrow, broad medially; anterior margin rounded; posterolateral margin lightly sclerotized; sternum IX without modification. Tergum X incompletely fused to tergum IX with membrane or lightly sclerotized region ventrolaterally; dorsomesal margin with single, downturned, elongate process; dorsolateral margin irregular and setose; ventrolateral margin with paired, broad flange-like setose process consisting of small upper lobe and larger subtriangular lower lobe. Inferior appendages present as apically bifid, setose process produced mesally, broadest at base and fused to phallobase ventrobasally, with 2 pairs of small digitate lobes ventrolaterally, each bearing a seta. Parameres present, paired, inserted in membranous lobe, arising laterally from endotheca, sclerotized and rod-like, long, sinuous, directed outward and posteriorly, apex pointed. Phallobase reduced, lightly sclerotized. Phallicata forming a long sclerotized dorsal sheath extending from phallobase, broadest basally, narrowed slightly, distal portion curving dorsally. Endophallus membranous, enlarged and convoluted when invaginated, with 1 tubular upper lobe and 1 smaller lower lobe.

**Figure 23.**
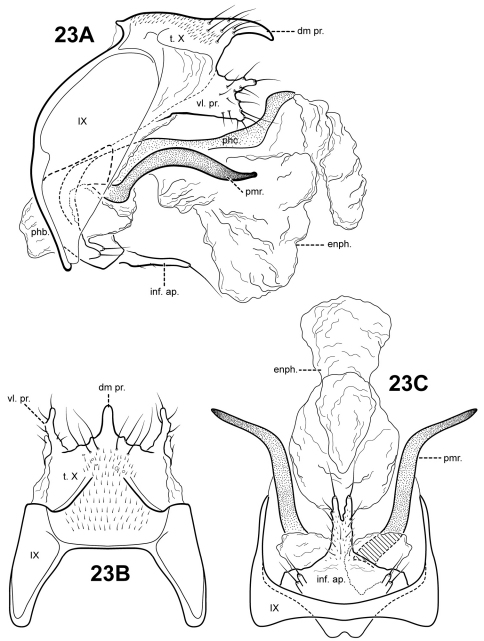
*Itauara stella* sp. n. (UMSP000052589). Male genitalia (**A**) lateral; (**B**) dorsal; (**C**) ventral. Abbreviations: dm. pr. = dorsomesal process; enph. = endophallus; inf. ap. = inferior appendage process; phb. = phallobase; phc. = phallicata; pmr . = paramere; t. X = tergum X; vl. pr. = ventrolateral process.

#### Material examined.

**Holotype male: BRAZIL: Sao Paulo:** Estação Biológica Boraceia: Rio Venerando & tribs, 23°39'11"S, 045°53'25"W, 850 m, 18–21.ix.2002 (Blahnik, Prather, Melo, Froehlich, Silva) (UMSP000052589) (MZUSP)

**Paratypes: BRAZIL: Sao Paulo:** same data as holotype — 9 males, 9 females (UMSP).

#### Etymology.

We are delighted to name this species for the senior author’s daughter, Stella Claire Thompson.

### Itauara tusci

Robertson & Holzenthal sp. n.

urn:lsid:zoobank.org:act:8080F8F1-4471-4A27-A919-65C247501BDD

http://species-id.net/wiki/Itauara_tusci

Fig. 24A–C 

#### Description. 

This species is very similar to *Itaura rodmani* sp. n., which also has very long, upturned, tusk-like parameres and a strongly upturned phallicata. The 2 species are separated based on the shape of the phallicata process, which is pointed and blade-like in *Itaura tusci* and rounded or blunt in *Itaura rodmani*. The phallicata of *Itaura tusci* is more lightly sclerotized than *Itaura rodmani*, especially at the distal portion. Additionally, in *Itaura tusci*, the dorsomesal margin of tergum X is irregular, with several small setose processes, whereas in *Itaura rodmani*, the dorsomesal margin is rather smooth and triangular. The ventrolateral processes of the 2 species also differ: *Itaura tusci* has a small upper and more elongate lower process; *Itaura rodmani* has a single, short, digitate process. *Itauara blahniki* also has upturned, tusk-like parameres, but is easily distinguished from *Itaura tusci* based on differences in tergum X and the phallicata.

Adult. Body, wings, and appendages pale or tawny brown, often intermingled with rufous or golden hairs, tibia and tarsi tawny brown. Wings with conspicuous white spot at the arculus. Forewing slightly broader past anastomosis, but with margins nearly parallel, apex rounded. Forewing venation incomplete, with apical forks I, II, and III present; Sc and R1 distinct along their entire lengths; fork I sessile; fork II petiolate, stem shorter than fork; fork III petiolate, stem about the same length as fork; Cu1 complete, reaching wing margin; Cu1 and Cu2 intersecting near anastomosis; row of erect setae present along Cu2; A3 absent; crossveins forming a relatively linear transverse cord; discoidal cell longer than Rs vein. Hind wing margins nearly parallel, tapering only slightly past anastomosis; apical forks II, III, and V present; Sc and R1 fused basally; A2 absent. Tibial spurs 1,4,4, foretibial spur extremely reduced and hairlike. Sixth sternal process thumb-like, apex attenuate and pointed, associated with weak oblique apodeme posteriorly.

Male genitalia. Preanal and inferior appendages absent. Segment IX dorsally narrow, broad medially and ventrally; anterior margin rounded; posterolateral margin membranous or very lightly sclerotized; sternum IX without modification. Tergum X incompletely fused to tergum IX with membrane or lightly sclerotized region ventrolaterally; dorsomesal margin slightly produced with several small irregular setose processes; dorsolateral margin with paired small, slightly down-turned, setose process; ventrolateral margin with 2 pairs of processes, the upper a small lobe-like process, the lower an elongate finger-like process bearing a few setae. Parameres present, paired, arising ventrobasally from fused endotheca and phallobase, sclerotized and rod-like, tusk-like, strongly curving upward, apex pointed. Phallobase reduced, lightly sclerotized with phallic shield. Phallicata forming a long lightly sclerotized dorsal sheath extending from phallobase, rugous distally, strongly curving upward with apex directed anterodorsally, with pair of broad, sclerotized blade-like lateral flanges, apex pointed and directed posteriorly. Endophallus membranous, enlarged and convoluted when invaginated, with 1 upper and 1 lower lobe.

**Figure 24.**
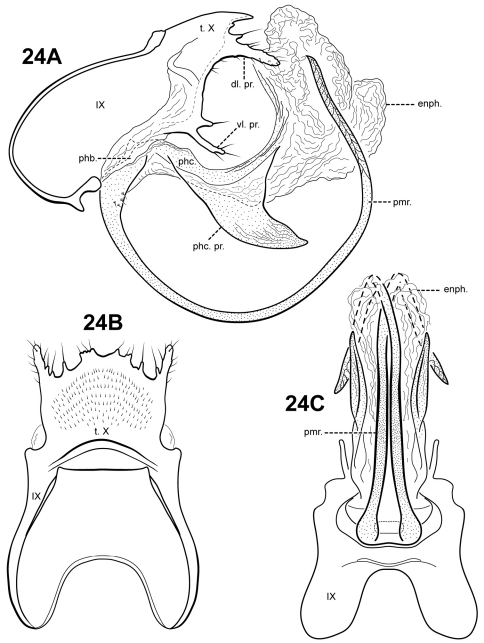
*Itauara tusci* sp. n. (UMSP000070932). Male genitalia: (**A**) lateral; (**B**) dorsal; (**C**) ventral. Abbreviations: dl. pr. = dorsolatera; process; enph. = endophallus; phb = phallobase; phc. = phallicata; phc. pr. phallicata process; pmr . = paramere; t. X = tergum X; vl. pr. = ventrolateral process.

#### Material examined.

 **Holotype male: BRAZIL: Rio de Janeiro:** Rio das Flores, Macaé de Cima, 10 km SE Mury, 1000 m, 9.iii.2002 (Holzenthal, Blahnik, Paprocki, Prather) (UMSP000070932) (MZUSP).

**Paratypes: BRAZIL: Rio de Janeiro:** same data as holotype — 4 males, 26 females (UMSP); Rio Macaé, Macaé de Cima, 22°23'41"S, 042°30'08"W, 1000 m, 8.iii.2002 (Holzenthal, Blahnik, Paprocki, Prather) — 2 males, 10 females (UMSP); Encontro dos Rios (Macaé/Bonito), 6 km S Lumiar, 22°23'29"S, 042°18'42"W, 600 m, 10.iii.2002 (Holzenthal, Blahnik, Paprocki, Prather) — 64 males, 145 females (MZUSP, UMSP).

#### Etymology.

The name *tusci* is derived from the Old English word for tusk, and refers to the extremely long parameres of this species.

### Itauara unidentata

Robertson & Holzenthal sp. n.

urn:lsid:zoobank.org:act:63403AB7-370C-4DA2-8628-C630D0C9C1E6

http://species-id.net/wiki/Itauara_unidentata

Fig. 2C 25A–C 

#### Description. 

This species can be diagnosed by its large, tooth-like paramere process, and broad inferior appendage process. It is most similar to *Itaura bidentata* sp. n., which has a similarly shaped tergum X, dorsomesal spine, and apical sclerites. The 2 species can be separated by their paramere processes; in *Itaura unidentata* the paramere consists of a single large tooth-like process, whereas in *Itaura bidentata*, the paramere process is bifid. *Itauara amazonica* also has a dorsomesal spine, but can be distinguished from *Itaura unidentata* by the simple shape of tergum X and parameres. Itauara *spiralis* sp. n., has a similarly shaped tergum X, but is easily distinguished from *Itaura unidentata* by differences in the shape of the inferior appendage process, parameres, and phallicata.

Adult. Body, wings, and appendages pale or tawny brown in alcohol. Forewing slightly broader past anastomosis, but with margins nearly parallel, apex subacute. Forewing venation incomplete, with apical forks I, II, III, and IV present; Sc and R1 distinct along their entire lengths; fork I sessile; fork II sessile; fork III petiolate, stem longer than fork; fork IV petiolate, stem slightly shorter than fork; Cu1 complete, reaching wing margin; Cu1 and Cu2 intersecting near anastomosis; row of erect setae present along Cu2; A3 absent; crossveins forming a relatively linear transverse cord; discoidal cell longer than Rs vein. Hind wing narrow and slightly scalloped past anastomosis; apical forks II and V present; Sc and R1 fused basally; A2 absent. Tibial spurs 1,4,4, foretibial spur extremely reduced and hairlike. Sixth sternal process short and digitate, apex attenuate and pointed, associated with strong oblique apodeme posteriorly.

Male genitalia. Preanal appendages absent. Segment IX dorsally and ventrally narrow, broad medially; anterior margin rounded; posterolateral margin membranous or very lightly sclerotized; sternum IX without modification. Tergum X incompletely fused to tergum IX with membrane or lightly sclerotized region ventrolaterally; dorsomesal margin straight, without processes; dorsolateral margin with paired elongate, down-turned, finger-like process; ventrolateral margin with paired, broad flange-like setose process consisting of several small irregular lobes. Inferior appendages present as single, broad, irregular setose process, broadest basally, fused to phallobase ventrobasally, bearing a single pair of small digitate lobes ventrolaterally, each bearing a seta. Parameres present, paired, arising laterally from endotheca, strongly sclerotized, large tooth-like process, curving ventrally and outward, apex pointed. Phallobase reduced, lightly sclerotized dorsally, laterally membranous, with 2 irregular and elongate sclerites arising basolaterally. Phallicata forming a short slerotized dorsal sheath with an elongate dorsomesal spine arising posteriorly to phallobase.

**Figure 25.**
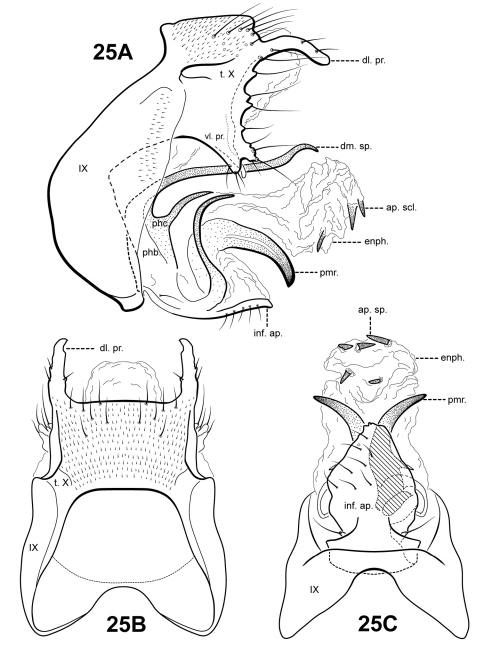
*Itauara unidentata* sp. n. (UMSP000118535). Male genitalia (**A**) lateral; (**B**) dorsal; (**C**) ventral. Abbreviations: ap. sp. = apical spine; dl. pr. = dorsolateral process; dm. sp. = dorsomesal spine; enph. = endophallus; inf. ap. = inferior appendage process; phb. = phallobase; phc. = phallicata; pmr . = paramere; t. X = tergum X; vl. pr. = ventrolateral process.

#### Material examined.

 **Holotype male: GUYANA: Kanuku Mountains:** Kumu River & Falls, 03°15'54"N, 059°43'30"W, 28–30.iv.1995 (W.N. Mathis) (UMSP000118535) (NMNH).

**Paratypes:** **GUYANA: Kanuku Mountains:** same data as holotype, (W.N. Mathis) — 1 female (NMNH); same, (O.S. Flint) — 1 male (NMNH).

#### Etymology.

The name *unidentata* is suggested by the single tooth-like paramere process.

##### Key to males of Itauara

In most cases, it should be possible to identify most species by simple comparisons to illustrations and reference to the species diagnoses and descriptions. The following key is meant to help the user focus on male genitalic features most useful in identifying species and should be used in conjunction with the provided illustrations and descriptions.

**Table:** 

1	Inferior appendages present as single, sometimes apically bifid, setose process, fused to phallobase ventrobasally (Figs 4–6, 10, 13, 14, 16–18, 22, 23, 25)	2
–	Inferior appendages absent (Figs 7–9, 11, 12, 15, 19–21, 24)	13
2(1)	Inferior appendage process relatively short and broad (Figs 4C, 6C, 13C, 14C, 16C, 22C, 25C)	3
–	Inferior appendage process relatively narrow and elongate (Figs 5D, 10C, 17C, 18C, 23C)	9
3(2)	Tergum X dorsolateral margin with paired, very long, finger-like processes; phallicata with elongate dorsomesal spine (Figs 6, 22, 25)	4
–	Tergum X dorsolateral margin without paired long, finger-like processes; phallicata without dorsomesal spine (Fig. 4, 13, 14, 16)	6
4(3)	Inferior appendage process bifid apically; endophallus without apical sclerites; parameres rather elongate, spiral-shaped, curving nearly 360 degrees (Fig. 22)	*Itauara spiralis* sp. n.
–	Inferior appendage process not bifid; endophallus with apical sclerites; parameres rather broad and tooth-like, not spiral-shaped (Figs 6, 25)	5
5(4)	Parameres bifid (Fig. 6)	*Itauara bidentata* sp. n.
–	Parameres not bifid (Fig. 25)	*Itauara unidentata* sp. n.
6(3)	Tergum X with elongate, attenuate, downturned dorsomesal process (Figs 4A–B, 13A–B, 16A–B)	7
–	Tergum X dorsomesal margin bifid (Fig. 14)	*Itauara jamesii* sp. n.
7(6)	Inferior appendage process bifid apically (Fig. 4C)	*Itauara alexanderi* sp. n.
–	Inferior appendage process not bifid (Figs 13C, 16C)	8
8(7)	Parameres extremely sinuous, not forked; endophallus with large, bifid apical processes; tergum X ventrolateral margin with small, subtriangular setose process (Fig. 13)	*Itauara guyanensis* sp. n.
–	Parameres forked, not sinuous; endophallus entirely membranous without apical processes or sclerites; tergum X ventrolateral margin with broad, irregular, flange-like setose process (Fig. 16)	*Itauara lucinda* sp. n.
9(2)	Tergum X dorsomesal margin divided apicomesally, slightly notched, or with large, prominent bifid process; inferior appendage process not bifid (Figs 5, 17, 18)	10
–	Tergum X dorsomesal margin not divided apicomesally, bifid, slightly notched, or with large, prominent bifid process; inferior appendage process bifid apically (Figs 10, 23)	12
10(9)	Parameres curving upward or tusk-like; phallicata with elongate, apically bifid, dorsomesal spine (Fig. 5)	*Itauara amazonica* (Flint 1971)
–	Parameres not curving upward or tusk-like; phallicata without dorsomesal spine (Figs 17, 18)	11
11(10)	Parameres spiral-shaped or ram-like, curving nearly 360 degrees; endophallus largely membranous, without apical sclerite (Fig. 17)	*Itauara ovis* sp. n.
–	Parameres nearly straight; endophallus lightly sclerotized, with small apical sclerite (Fig. 18)	Itauara peruensis sp. n.
12(9)	Tergum X with elongate, attenuate, downturned dorsomesal process (Fig. 23)	*Itauara stella* sp. n.
–	Tergum X with large, blunt, dorsomesal process, elongate in dorsal view, subtriangular in lateral view (Fig. 10)	*Itauara emilia* sp. n.
13(1)	Phallicata with paired lateral flanges or processes (Figs 9A, 11A, 12B, 19B, 20B, 24A)	14
–	Phallicata without paired lateral flanges or processes (Figs 7A, 8A, 15A, 21A)	19
14(13)	Tergum X dorsomesal margin irregular, with several small setose processes (Figs 9B, 24B)	15
–	Tergum X dorsomesal margin not irregular (Figs 11A–B, 12B–C)	16
15(14)	Parameres curving upward, tusk-like; endophallus without apical processes or sclerites (Fig. 24)	*Itauara tusci* sp. n.
–	Parameres arcuate, curving downward; endophallus with tooth-like apical sclerite (Fig. 9)	*Itauara charlotta* sp. n.
16(14)	Tergum X with elongate, attenuate, downturned dorsomesal process (Figs 11A–B, 12B–C)	17
–	Tergum X without elongate dorsomesal processes (Figs 19B–C, 20B–C)	18
17(16)	Parameres sinuous; phallicata very lightly sclerotized basally, rugous or membranous distally (Fig. 12)	*Itauara guarani* (Angrisano 1993)
–	Parameres arcuate, curving downward; phallicata entirely sclerotized (Fig. 11)	*Itauara flinti* sp. n.
18(16)	Parameres curving upward, tusk-like; phallicata strongly curved medially, directed anterodorsally (Fig. 20)	*Itauara rodmani* sp. n.
–	Parameres sinuous; phallicata nearly straight, distal portion slightly upturned (Fig. 19)	*Itauara plaumanni* (Flint 1974)
19(13)	Sternum IX bearing 2 pairs of extremely elongate, seta-like processes; parameres vestigial, consisting only of a pair of small, digitate setose lobes arising ventrolaterally from endotheca (Fig. 8)	*Itauara brasiliana* (Mosely 1939)
–	Sternum IX without modification; parameres prominently present (Figs 7, 15, 21)	20
20(19)	Tergum X with elongate, attenuate, slightly downturned dorsomesal process; parameres curving upward, tusk-like (Fig. 7)	*Itauara blahniki* sp. n.
–	Tergum X without elongate dorsomesal processes; parameres not curving upward or tusk-like (Figs 15, 21)	21
21(20)	Tergum X dorsomesal margin blunt; parameres bent basally at nearly 90 degree angle, directed dorsally; phallicata sclerotized, not continuous from phallobase (Fig. 15)	*Itauara julia* sp. n.
–	Tergum X dorsomesal margin roof-like, strongly produced; parameres arcuate, curving downward; phallicata very lightly sclerotized basally, more membranous and rugous distally, continuous from phallobase (Fig. 21)	*Itauara simplex* sp. n.

## Supplementary Material

XML Treatment for Itauara

XML Treatment for Itauara alexanderi

XML Treatment for Itauara amazonica

XML Treatment for Itauara bidentata

XML Treatment for Itauara blahniki

XML Treatment for Itauara brasiliana

XML Treatment for Itauara charlotta

XML Treatment for Itauara emilia

XML Treatment for Itauara flinti

XML Treatment for Itauara guarani

XML Treatment for Itauara guyanensis

XML Treatment for Itauara jamesii

XML Treatment for Itauara julia

XML Treatment for Itauara lucinda

XML Treatment for Itauara ovis

XML Treatment for Itauara peruensis

XML Treatment for Itauara plaumanni

XML Treatment for Itauara rodmani

XML Treatment for Itauara simplex

XML Treatment for Itauara spiralis

XML Treatment for Itauara stella

XML Treatment for Itauara tusci

XML Treatment for Itauara unidentata
